# MIR137 polygenic risk for schizophrenia and ephrin-regulated pathway: Role in lateral ventricles and corpus callosum volume

**DOI:** 10.1016/j.ijchp.2024.100458

**Published:** 2024-04-09

**Authors:** G.A.M. Blokland, N. Maleki, J. Jovicich, R.I. Mesholam-Gately, L.E. DeLisi, J.A. Turner, M.E. Shenton, A.N. Voineskos, R.S. Kahn, J.L. Roffman, D.J. Holt, S. Ehrlich, Z. Kikinis, P. Dazzan, R.M. Murray, J. Lee, K. Sim, M. Lam, S.M.C. de Zwarte, E. Walton, S. Kelly, M.M. Picchioni, E. Bramon, N. Makris, A.S. David, V. Mondelli, A.A.T.S. Reinders, E. Oykhman, D.W. Morris, M. Gill, A.P. Corvin, W. Cahn, N. Ho, J. Liu, R.L. Gollub, D.S. Manoach, V.D. Calhoun, S.R. Sponheim, S.L. Buka, S. Cherkerzian, H.W. Thermenos, E.W. Dickie, S. Ciufolini, T. Reis Marques, N.A. Crossley, S.M. Purcell, J.W. Smoller, N.E.M. van Haren, T. Toulopoulou, G. Donohoe, J.M. Goldstein, M.S. Keshavan, T.L. Petryshen, E.C. del Re

**Affiliations:** a Department of Psychiatry and Neuropsychology, School for Mental Health and Neuroscience, Faculty of Health, Medicine, and Life Sciences, Maastricht University, Netherlands; b Psychiatric and Neurodevelopmental Genetics Unit, Center for Genomic Medicine, Massachusetts General Hospital, Boston, MA, United States; c Department of Psychiatry, Massachusetts General Hospital, Boston, MA, United States; d Department of Psychiatry, Harvard Medical School, Boston, MA, United States; e Stanley Center for Psychiatric Research, Broad Institute of MIT and Harvard, Cambridge, MA, United States; f MGH/HST Athinoula A. Martinos Center for Biomedical Imaging, Massachusetts General Hospital, Charlestown, MA, United States; g Center for Mind/Brain Sciences (CIMeC), University of Trento, Trento, Italy; h Massachusetts Mental Health Center Public Psychiatry Division, Beth Israel Deaconess Medical Center, Boston, MA, United States; i Beth Israel Deaconess Medical Center, Harvard Medical School, Boston, MA, United States; j Department of Psychiatry, Cambridge Health Alliance, Cambridge, MA, United States; k Department of Psychiatry and Behavioral Health, The Ohio State University, Columbus, OH, United States; l Psychiatry Neuroimaging Laboratory, Department of Psychiatry, Brigham and Women's Hospital, Boston, MA, United States; m Department of Radiology, Brigham and Women's Hospital, Harvard Medical School, Boston, MA, United States; n Department of Psychiatry, Veterans Affairs Boston Healthcare System, Brockton, MA, United States; o Kimel Family Translational Imaging Genetics Laboratory, Department of Psychiatry, Research Imaging Centre, Campbell Family Mental Health Institute, Centre for Addiction and Mental Health, Faculty of Medicine, University of Toronto, Toronto, ON, Canada; p Department of Psychiatry and Institute of Medical Science, University of Toronto, Toronto, ON, Canada; q Brain Centre Rudolf Magnus, Department of Psychiatry, University Medical Centre Utrecht, Utrecht, The Netherlands; r Division of Psychological & Social Medicine and Developmental Neurosciences, Faculty of Medicine, Technische Universität Dresden, Dresden, Germany; s Institute of Psychiatry, Psychology, and Neuroscience, King's College London, London, United Kingdom; t National Institute for Health Research (NIHR) Mental Health Biomedical Research Centre at South London and Maudsley NHS Foundation Trust, London, United Kingdom; u Institute of Mental Health, Woodbridge Hospital, Singapore; v Analytical & Translational Genetics Unit, Center for Genomic Medicine, Massachusetts General Hospital, Boston, MA, United States; w Division of Psychiatry Research, The Zucker Hillside Hospital, Northwell Health, Glen Oaks, NY, USA; x Department of Psychology, University of Bath, Bath, United Kingdom; y Neuropsychiatric Genetics Research Group, Department of Psychiatry, Institute of Molecular Medicine, Trinity College Dublin, Dublin, Ireland; z Laboratory of NeuroImaging, Keck School of Medicine, University of Southern California, Los Angeles, CA, United States; aa Mental Health Neuroscience Research Department, UCL Division of Psychiatry, University College London, United Kingdom; ab Department of Neurology, Massachusetts General Hospital, Boston, MA, United States; ac Department of Neurology, Harvard Medical School, Boston, MA, United States; ad Division of Psychiatry, University College London, London, United Kingdom; ae Cognitive Genetics and Cognitive Therapy Group, Neuroimaging and Cognitive Genomics (NICOG) Centre and NCBES Galway Neuroscience Centre, School of Psychology and Discipline of Biochemistry, National University of Ireland, Galway, Ireland; af Genome Institute, Singapore; ag Tri-institutional Center for Translational Research in Neuroimaging and Data Science (TReNDS), Georgia State, Georgia Tech, Emory, Atlanta, GA, United States; ah Department of Psychiatry, University of Minnesota, Minneapolis, MN, United States; ai Department of Epidemiology, Brown University, Providence, RI, United States; aj Department of Medicine, Division of Women's Health, Brigham and Women's Hospital, Harvard Medical School, Boston, MA, United States; ak Department of Psychiatry, Brigham and Women's Hospital, Boston, MA, United States; al Division of Psychiatric Genomics, Departments of Psychiatry and Genetics and Genomic Sciences, Icahn School of Medicine at Mount Sinai, New York, NY, United States; am Department of Child and Adolescent Psychiatry/Psychology, Erasmus Medical Centre, Rotterdam, The Netherlands; an Department of Psychiatry, University Medical Centre Utrecht, Utrecht, The Netherlands; ao Department of Psychology & National Magnetic Resonance Research Center (UMRAM), Aysel Sabuncu Brain Research Centre (ASBAM), Bilkent University, Ankara, Turkey; ap Department of Psychiatry, Faculty of Medicine, National and Kapodistrian University of Athens, Athens, Greece; aq Department of Psychiatry, Icahn School of Medicine at Mount Sinai, New York, NY, United States; ar University of Pittsburgh Medical Center, Pittsburgh, PA, United States; as Trinity College Institute of Neuroscience, Trinity College Dublin, Dublin, Ireland

**Keywords:** Corpus callosum, Lateral ventricles, MIR137, Polygenic risk score, Schizophrenia, Ephrin pathway

## Abstract

*Background/Objective.* Enlarged lateral ventricle (LV) volume and decreased volume in the corpus callosum (CC) are hallmarks of schizophrenia (SZ). We previously showed an inverse correlation between LV and CC volumes in SZ, with global functioning decreasing with increased LV volume. This study investigates the relationship between LV volume, CC abnormalities, and the microRNA MIR137 and its regulated genes in SZ, because of MIR137’s essential role in neurodevelopment. *Methods*. Participants were 1224 SZ probands and 1466 unaffected controls from the GENUS Consortium. Brain MRI scans, genotype, and clinical data were harmonized across cohorts and employed in the analyses. *Results.* Increased LV volumes and decreased CC central, mid-anterior, and mid-posterior volumes were observed in SZ probands. The MIR137-regulated ephrin pathway was significantly associated with CC:LV ratio, explaining a significant proportion (3.42 %) of CC:LV variance, and more than for LV and CC separately. Other pathways explained variance in either CC or LV, but not both. CC:LV ratio was also positively correlated with Global Assessment of Functioning, supporting previous subsample findings. SNP-based heritability estimates were higher for CC central:LV ratio (0.79) compared to CC or LV separately. *Discussion.* Our results indicate that the CC:LV ratio is highly heritable, influenced in part by variation in the MIR137-regulated ephrin pathway. Findings suggest that the CC:LV ratio may be a risk indicator in SZ that correlates with global functioning.

## Introduction

Schizophrenia (SZ) is a major neurodevelopmental disorder affecting 1 % of the population worldwide (Velligan & Rao, 2023). Clinical manifestations include psychotic symptoms, cognitive, global and social dysfunctions. Increases in the volume of the lateral ventricles (LV) were among the first identified abnormalities in SZ (Johnstone et al., 1976) and since then, increases in LV volume remain among the most reliable volumetric abnormalities reported in SZ (del Re et al., 2016a; Kelly et al., 2018; Kempton et al., 2010; Konishi et al., 2018; Lizano et al., 2019; van Erp et al., 2018). Increases are found throughout the course of SZ (del Re et al., 2016a; Nakamura et al., 2007). SZ-related abnormalities in the corpus callosum (CC) have also been described, specifically reduced midsagittal surface area in chronic and first episode patients with psychosis (Arnone et al., 2008; Keshavan et al., 2002), and reduced central CC volume (del Re et al., 2016a). However, few studies have looked at the relationship between LV and CC in the same investigation in SZ, a surprising fact as both LV and CC abnormalities are hallmark features of SZ and anatomically interlinked.

In SZ, markedly abnormal volume of the LV indexes poor-outcome, unremitting SZ, characterized by severe disturbances in social functioning, negative symptoms and cognitive deterioration (Cahn et al., 2006; DeLisi et al., 1992; Mitelman & Buchsbaum, 2007). Also of note, within 1 year of psychosis onset, enlarged LV indexes greater change in the Brief Psychosis Rating Scale withdrawal-retardation score (Nakamura et al., 2007) while in early SZ (up to three years after psychotic outbreak), LV changes index longitudinal change in global functioning (del Re et al., 2016a). Reduced connectivity based on tract segmentation of the CC has also been reported in SZ (Kubicki et al., 2008) as has fiber geometry alterations in the CC in SZ, suggesting a transcallosal misconnection (Whitford et al., 2011). In investigating white matter anomalies, fractional anisotropy (FA), a measure of the direction of diffusion in the brain, shows that decreased FA of the CC indexes higher symptomatology (del *Re* et al., 2019). Data from our team have also shown that FA of the central CC inversely correlates with LV volume in SZ (del *Re* et al., 2019) (see also (Lett et al., 2013)). Mild ventriculomegaly detected prenatally has also been associated with larger mean and radial diffusivity and reduced FA of the CC, which persist in neonates (Gilmore et al., 2008). There is also evidence that prenatal LV width significantly and negatively correlates with a measure of cognitive development at a mean age of 21 months (Bloom et al., 1997), predicting postnatal neurocognitive development.

The heritability of SZ estimated from twin studies is relatively high (∼81 %; Baselmans et al. (2021)). Nonetheless, the neural mechanisms underlying SZ are still largely unknown. Genome-wide association studies (GWAS) have contributed insight into the pathophysiology of SZ (Bergen & Petryshen, 2012). Several neurodevelopmental disorders show aberrations in genes associated with risk of SZ and/or bipolar disorder in GWAS (Wijmenga & Zhernakova, 2018). Here we examine MIR137 and MIR137-regulated pathways for the genetic association with LV and CC measures. The MIR137 SZ associated variant (single nucleotide polymorphism; SNP) is one of the strongest SZ associated SNPs from the GWAS by the Psychiatric Genomic Consortium (PGC) (Schizophrenia Working Group of the Psychiatric Genomics Consortium, 2014; Trubetskoy et al., 2022). The rationale for including this gene in the current study is that as a microRNA, MIR137 is a short non-coding RNA molecule that functions to regulate the expression levels of other genes. MIR137 regulates gene transcription, with a prime role in development (Miller et al., 2012), in adult neural stem cell maturation and migration in the subventricular zone, which is in close proximity to the LV. This gene further regulates gliogenesis, which is crucial to white matter neurodevelopment (Wright et al., 2013), underscoring the involvement of both ventricular and white matter development and its potential role in SZ.

Homozygous MIR137-knockout mice do not survive the embryonic stage, while heterozygotes seemingly develop normally, indicating MIR137’s essential role in development, with biological compensatory mechanisms in place (Crowley et al., 2015). Partial MIR137 loss in mice causes repetitive behavior, lack of sociability and impaired learning (Yan et al., 2019), while overexpression in transgenic mice causes behavioral deficits and transcriptome profiles related to SZ (Arakawa et al., 2019). Gene sets of potential MIR137 targets (Hill et al., 2014) are enriched with variants associated with SZ risk, including TCF4, involved with enlargement of LV, hypoplasia of CC and mental delays (Goodspeed et al., 2018; Kim et al., 2020; Zollino et al., 2019; Zweier et al., 2007); cortical expansion and neuronal differentiation (Tomasello et al., 2022); GRIN2A, involved in the NMDA receptor pathway (Gandal et al., 2012; Harrison & Bannerman, 2023) and possibly associated with negative symptoms (Coyle & Tsai, 2004; Javitt et al., 1994; Poltavskaya et al., 2023); CACNA1C, a risk factor also for bipolar disorder and major depression (Green et al., 2010; Wang et al., 2023); and ZNF804A, central to cognition (del *Re* et al., 2014; Novaes de Oliveira Roldan et al., 2023). Many of these MIR137-regulated and SZ-associated risk genes demonstrate genetic overlap with syndromes such as Pitt-Hopkins (TCF4) (Jung et al., 2018; Peippo & Ignatius, 2012; Teixeira et al., 2021), in which LV enlargement, CC hypoplasia and mental delays are present.

The frequent co-occurrence of LV and CC changes in such syndromes and in SZ, suggests that changes in the ratio of CC and LV volume may be a sensitive indicator of developmental alterations in the brain. Deviations from typical ratios may indicate disruptions in normal neurodevelopment, which could be influenced by genetic factors such as MIR137. By assessing the ratio in addition to absolute volumes, we may be able to detect subtle variations and genetic associations that may be missed when considering each structure independently.

Therefore, we combined two approaches, a pathway- and polygenic score-based approach, to determine the impact of MIR137-regulated genes on LV and CC measures, including their ratios. Whereas polygenic scoring captures great variability in the sample and provides a polygenic score (PGS) for each subject, this approach does not identify underlying biological pathways. The pathway analyses, on the other hand, can implicate specific gene networks involved in disease pathogenesis. MIR137-regulated pathways, enriched with MIR137-regulated SZ risk variants, include axonal guidance signaling, ephrin receptor signaling, long-term potentiation (LTP), pre-synaptic plasticity, and protein kinase A (PKA) signaling (Kwon et al., 2013; Wijmenga & Zhernakova, 2018).

Here, we present novel SNP-based heritability of LV, CC and of CC:LV ratio utilizing the Massively Expedited Genome-wide Heritability Analysis (MEGHA), an accurate genome-wide SNP methodology for heritability estimates of phenotypes (Ge et al., 2015). We rely on data from the GENUS Consortium (Blokland et al., 2018), including 1224 SZ patients and 1466 healthy controls (HC), for volumetric and genetic analysis of the CC and LV and their relationship to symptoms and general functioning.

Based on our previous studies, we hypothesize that MIR137, and its regulated genes, are associated with LV and CC abnormalities in SZ that underlie symptoms and impaired functioning.

## Methods

### Participants: genus data collection

The GENUS Consortium is a collaborative SZ neurogenetics project (Blokland et al., 2018). Eleven sites worldwide have contributed MRI data, along with GWAS, cognitive, and symptom data, from SZ patients (*n* = 1224) and HC (*n* = 1466), and familial high-risk individuals (FHR; *n* = 256). Inclusion and exclusion criteria by cohort have been described elsewhere (Blokland et al., 2018). The lead principal investigator for each sample verified approval from their institutional ethics committee for sharing human subject data. All research participants provided written informed consent (or legal guardian consent and subject assent). Ethics approval for the GENUS Consortium study at the central site, including genotyping of DNA samples for several cohorts, was obtained from the Partners Healthcare (USA, now Mass General Brigham) Institutional Review Board. All data were anonymized prior to transfer to the central site. A non-WMO declaration (in line with the Medical Research Involving Human Subjects Act) from the Medical Ethical Review Committee at MUMC+ was obtained to continue the research in Maastricht, the Netherlands, with fully deidentified data.

### MRI processing and standardization

To maximize compatibility of imaging data across sites, all scans were reprocessed using the same processing pipeline. Quality checks of 12 T1-weighted scans (partial volume coverage, wrap-around and motion artifacts, etc.) from each site were employed to determine scan quality. Scans were masked to separate brain from surrounding tissue using manual tracing, or using a novel automated multi-atlas brain segmentation (MABS) technique that performs similar to gold-standard manual tracing (del Re et al., 2016b). FreeSurfer version 5.3 (Fischl, 2012) was used to extract region-of-interest gray and white matter volumes using the Desikan-Killiany atlas (Desikan et al., 2006; Fischl et al., 2004). Ratios of CC subregion volumes relative to LV volumes were calculated from the FreeSurfer-extracted volumes.

Subsequently, Z scores, comparable across samples, were calculated (*Z_i_* = (*x*_i_ - *M_HC_) / SD_HC_*, where *i* represents the individual, and *M_HC_* and *SD_HC_* represent the within-cohort control mean and standard deviation), thereby providing a single consistent variable for FreeSurfer measures. Outliers >= 6 SD from the mean were removed, and remaining outliers >= 4 SD from the mean were winsorized.

### Genotype data

Genome-wide SNP genotype data were obtained by each site using different SNP arrays (See Blokland et al. (2018) for details). Quality control (QC) analyses of raw genotype data were carried out using PLINK 1.9 (Chang et al., 2015; Purcell et al., 2007). To increase genome coverage and generate a uniform dataset from the multiple genotyping arrays used for the samples, genotypes were imputed to the 1000 Genomes Phase III reference panel (The 1000 Genomes Project Consortium et al., 2015), using IMPUTE2 software with pre-phasing by SHAPEIT2 (Delaneau et al., 2013; Howie et al., 2012) using the Rapid Imputation and COmputational PIpeLIne for GWAS (RICOPILI) (Lam et al., 2020).

### Genetic analyses

*Pathway Polygenic Risk Scoring and Association Analyses.* Polygenic Risk Scores (PRSs) were calculated using the PGC SZ GWAS (Schizophrenia Working Group of the Psychiatric Genomics Consortium, 2014) as the discovery set, due to the availability of leave-one-out summary statistics. For pathway PRS, we utilized previously described MIR137 pathways: Ephrin receptor signaling, synaptic long-term potentiation, PKA signaling and axonal guidance signaling (see (Wright et al., 2015) for a description of where and how the gene sets were defined). Additionally, as described in Cosgrove et al. (2017), we constructed the MIR137 downstream pathway based on the set of 1016 genes whose expression was identified as being altered by MIR137 manipulation (Hill et al., 2014); 831 of these genes could be unambiguously mapped to the autosomes and this gene set was used to generate PRS.

To test the aggregate effect of each pathway, a score was calculated for each subject applying published methods (Purcell et al., 2009; Terwisscha van Scheltinga et al., 2013; Walton et al., 2013) using PLINK 1.9 (Chang et al., 2015). For each gene in a pathway, all SNPs within 20 kb of the gene were identified. For each of these SNPs, a score was assigned to the individual that depends on whether the individual carries 0, 1, or 2 alleles associated with SZ risk in the PGC GWAS meta-analysis (Schizophrenia Working Group of the Psychiatric Genomics Consortium, 2014) weighted by the log of the PGC GWAS allele odds ratios. Summing the scores for all SNPs in a pathway results in an aggregate score for each individual. The overall polygenic risk identified in the PGC SZ GWAS meta-analysis was examined using a similar approach, calculating a score for each individual using all SNPs associated with SZ. As customary for polygenic risk scoring, scores were calculated for different sets of SNPs that surpass increasingly more stringent statistical thresholds in the PGC GWAS. These analyses were considered one test for multiple testing correction. See Supplementary Table 1 for a summary of the composition of the risk scores.

Linear regression analyses per cohort were performed using R version 3.5.3 to test association between the PGC-SZ2-defined PRS (Schizophrenia Working Group of the Psychiatric Genomics Consortium, 2014), the MIR137 gene, 5 MIR137 biological pathways, MIR137 targets, and 7 primary neural phenotypes: Volume of LV, volumes of 5 CC sections and total CC, and (6) ratios of CC (sections) to LV. Covariates used to adjust for potential biases include age, sex, age^2^, age × sex, age^2^ × sex, total brain volume, scanner dummies for multi-scanner cohorts, and 4 multi-dimensional scaling (MDS) ancestry principal components against population stratification (obtained using PLINK). Linear regression results from the individual cohorts (betas and standard errors) were submitted to inverse-variance weighted meta-analysis using the R ‘metafor’ package version 4.3–7 (Viechtbauer, 2010). False Discovery Rate (FDR) correction for the effective number of tests performed, based on the correlations between phenotypes, and correlations between PRSs (Derringer, 2018; Nyholt, 2004) was applied (*p* = 0.05 / [1 SNP + 1 PRS + 5 pathways] × 7 CC and LV variables]). The ratio variables were not considered independent phenotypes for FDR, and the associations with PRS generated at different discovery GWAS thresholds were not considered independent tests.

Primary analyses were performed by analyzing SZ cases and HC together (European ancestry only), since genetic variation may have the same effect in healthy and diseased states, and to increase analytical power, owing to a larger sample size and greater phenotypic variance. Sensitivity association analyses were carried out for a few data configurations: cases, controls, and FHR individuals combined for European ancestry only; and for European + East Asian ancestry; cases and controls separately for European ancestry only, and for European + East Asian ancestry; males and females separately for European ancestry only, and for European + East Asian ancestry.

*SNP-based Heritability and Co-heritability.* SNP-based heritability of LV and CC was assessed using MEGHA (Ge et al., 2015). Using a linear mixed effects model, MEGHA uses the GWAS SNP association data for LV and CC volume to estimate how much of the variance in these measures is due to common genetic variants (SNPs). Covariates were as above.

### Clinical and cognitive measures

Symptom ratings based on the Positive and Negative Syndrome Scale (PANSS) (Kay et al., 1987) or Scales for the Assessment of Negative/Positive Symptoms (SANS/SAPS) (Andreasen, 1983, 1984), Global Assessment of Functioning (GAF) (American Psychiatric Association, 1994), current chlorpromazine equivalent antipsychotic medication dosage, age at onset, duration of illness in years, premorbid and current IQ were available. Premorbid IQ was estimated from single word reading tests, or the Wechsler Adult Intelligence Scale (WAIS) Vocabulary subtest. Current IQ was estimated based on one to eight WAIS subtests. See (Blokland et al., 2018) and Supplementary Materials for details on the medication and symptom data processing and tests used per cohort.

Due to significant deviations from normality for several clinical measures, Spearman partial correlations, adjusted for age and sex, were calculated between the clinical and cognitive measures and LV and CC neuroimaging phenotypes.

## Results

### Sample descriptives

*Demographic characteristics.* Significant differences in age, education level, premorbid and current IQ, male-female distributions, and ancestry distributions between SZ and HC diagnostic groups were observed (Table 1; see Supplementary Table 2 for comparisons with the FHR group).

**Table 1:** Demographic and clinical characteristics of the GENUS Consortium subset with MRI and genetic data that was included in the primary polygenic risk score (PRS) analyses. a.

	Patients		Controls		Statistic (F)	df	p e
	N	Mean ± SD (Range)	N	Mean ± SD (Range)			
Age (years)	720	35.2 ± 11.8 (16–76)	912	35.9 ± 12.9 (15–86)	1.1	1, 1630	0.29
Education Level (years) b	671	12.5 ± 2.7 (3–22)	702	14.5 ± 2.4 (7–21)	210.6	1, 1371	<0.001
Premorbid IQ	373	103±14.4 (56–145)	388	111.8 ± 10.7 (74.4–138)	92.1	1, 759	<0.001
Current IQ	365	99.4 ± 16.6 (58–155)	410	116.9 ± 14 (77–155)	256.4	1, 773	<0.001
Age at Onset (years)	633	23.5 ± 7.3 (9–58)	—	—	—	—	—
Illness Duration (years)	641	11.6 ± 11.6 (0–53)	—	—	—	—	—
PANSS Positive c	531	14±6 (7–41)	—	—	—	—	—
PANSS Negative c	528	13.7 ± 6.1 (7–42)	—	—	—	—	—
PANSS General	456	28.3 ± 11.7 (0–93)	—	—	—	—	—
Global Assessment of Functioning	117	14.6 ± 19.3 (0–103)	—	—	—	—	—
Chlorpromazine equivalent d current antipsychotic dose (mg)	120	20.5 ± 19.4 (0–103)	—	—	—	—	—
	**N**	**%**	**N**	**%**	**Statistic (χ^2^)**	**df**	**p**
Sex (female / male)	208 / 512	28.9 / 71.1	439 / 473	48.1 / 51.9	62.3	1	<0.001
Ancestral Population (EUR / EAS)	571 / 149	79.3 / 20.7	891 / 21	97.7 / 2.3	145.8	1	<0.001
Medication status (medicated / unmedicated / unknown)	503 / 116 / 101	69.9 / 16.1 / 14.0	—	—	—	—	—

Abbreviations: EAS = East Asian; EUR = European; IQ = Intelligence quotient; PANSS = Positive and Negative Syndrome Scale; SD = Standard Deviation.

a All available MRI data were used for standardization and covariate adjustment, regardless of availability of genetic data. See **Supplementary Table 2** for demographic and clinical characteristics for the full MRI dataset.

b Education level is measured in years from age 6, i.e., 12 years of education indicates high school completion (in most countries).

c Composite of PANSS and SANS/SAPS, calculated according to van Erp et al. (2014), is reported instead of scores from these scales separately, to increase sample size and reduce the multiple testing burden.

d Antipsychotic dose equivalent to 100 mg chlorpromazine.

e P-value from *t*-test (quantitative variables) or chi-squared test (categorical variables).

**Fig. 1:**
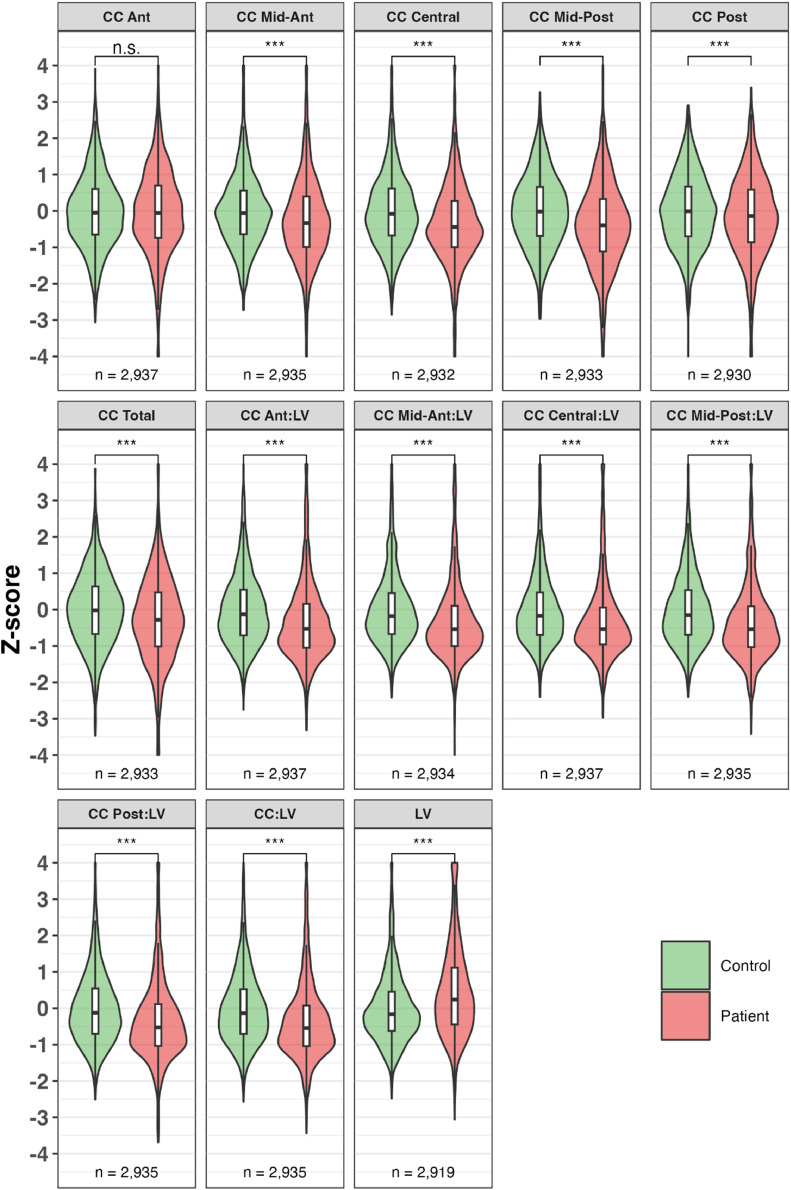
Covariate-adjusted standardized means and distributions of volumes of lateral ventricles and corpus callosum in the GENUS consortium.

*LV and CC.* All phenotypes displayed the expected SZ/HC differences (Fig. 1), with increased LV volumes in SZ (mean ∼ 0.5 SD above controls), and decreased CC volumes in SZ (mean ∼ 0.5 SD below controls). Differences for the FHR group are shown in Supplementary Figure 1.

### MIR137 pathway and SZ PRS associations

**Fig. 2:**
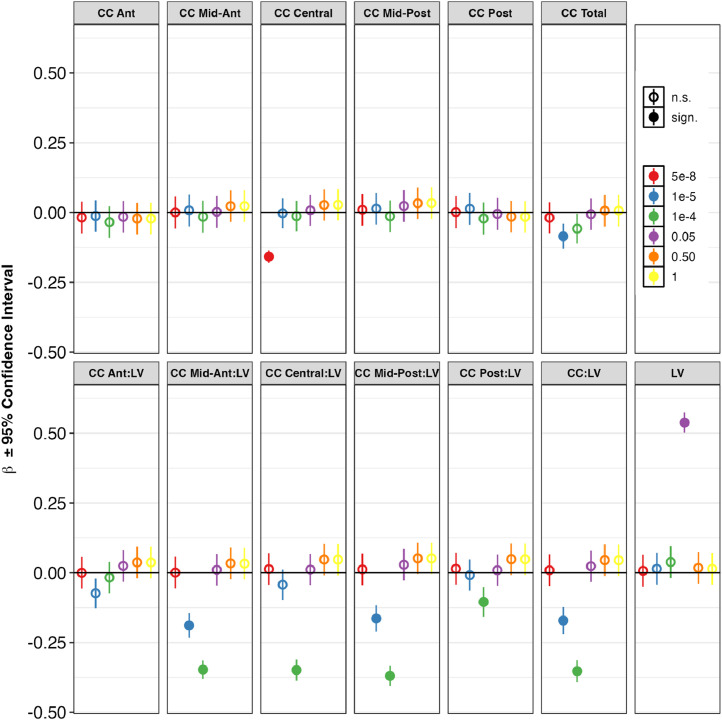
MIR137 ephrin pathway polygenic risk score (PRS) associations with volumes of lateral ventricles and corpus callosum, for patients and controls combined, excluding familial high-risk – European ancestry. For each phenotype, each of the six dots represents the beta value of the PRS calculated at a different discovery GWAS p-value threshold. A higher p-value means the PRS contains more SNPs. Abbreviations: Ant = Anterior, CC = corpus callosum, LV = lateral ventricles, Post = Posterior. * *p* < 0.05, FDR-corrected for 13 phenotypes and 7 PRSs.

All association results are shown in Supplementary Table 3. Association results in HC and SZ combined (European ancestry only) are shown in Fig. 2 and Supplementary Table 3a.

A MIR137 gene score alone explained on average 1.17 % (CC Mid-Posterior), 3.98 % (CC Mid-Anterior) and 5.19 % (LV) of the variance in the LV and CC volumes, but the associations did not reach significance (p_FDR_ > 0.05).

Among the 5 pathway PRS, the most significant associations were found between the *ephrin pathway* and multiple LV and CC volumes and ratios, specifically LV (β ± SE = 0.54 ± 0.02, average R^2^ across cohorts = 3.71 %); CC Mid-Ant:LV (β ± SE = −0.35 ± 0.02, average R^2^ = 2.65 %); CC Mid-Post:LV (β ± SE = −0.37 ± 0.02, average R^2^ = 2.93 %); CC Central:LV (β ± SE = −0.37 ± 0.02, average R^2^ = 3.12 %), and CC:LV (β ± SE = −0.35 ± 0.02, average R^2^ = 3.42 %). These effects were observed most clearly at a *p* < 1e-4 polygenic threshold, except for LV (0.05 threshold).

Other notable associations were between the *axon guidance pathway* and LV (β ± SE = 0.54 ± 0.02, average R^2^ = 4.87 %) and CC Posterior:LV (β ± SE = −0.15 ± 0.03, average R^2^ = 5.0 %); between the *LTP pathway* and LV (β ± SE = 0.10 ± 0.03, average R^2^ = 5.38 %); between the *PKA pathway* and CC Mid-Posterior (β ± SE = −0.09 ± 0.03, average R^2^ = 2.26 %); and between the *MIR137 targets* and CC Mid-Posterior (β ± SE = 0.10 ± 0.02, average R^2^ = 1.85 %).

Overall, CC (subregion):LV ratios and LV volume showed the highest percentages variance explained by the pathway PRS (6.24 % for LV), and the ephrin pathway was associated with most phenotypes. The ephrin pathway effects were observed most clearly at the *p* < 1e-5 and *p* < 1e-4 polygenic thresholds.

The PGC-SZ2 PRS was significantly associated with nearly all LV and CC volumes and ratios and explained on average 0.68 % (CC Central) to 5.7 % (LV) of the variance in the LV and CC volumes. These effects were observed most clearly at a *p* < 5e-8 polygenic threshold, although significant results were observed at all thresholds analyzed.

Results did not change significantly when including East Asian ancestry (Supplementary Table 3 g). Results for other data configurations (see above) are shown in Supplementary Tables 3b-3l

### MEGHA heritability

**Fig. 3:**
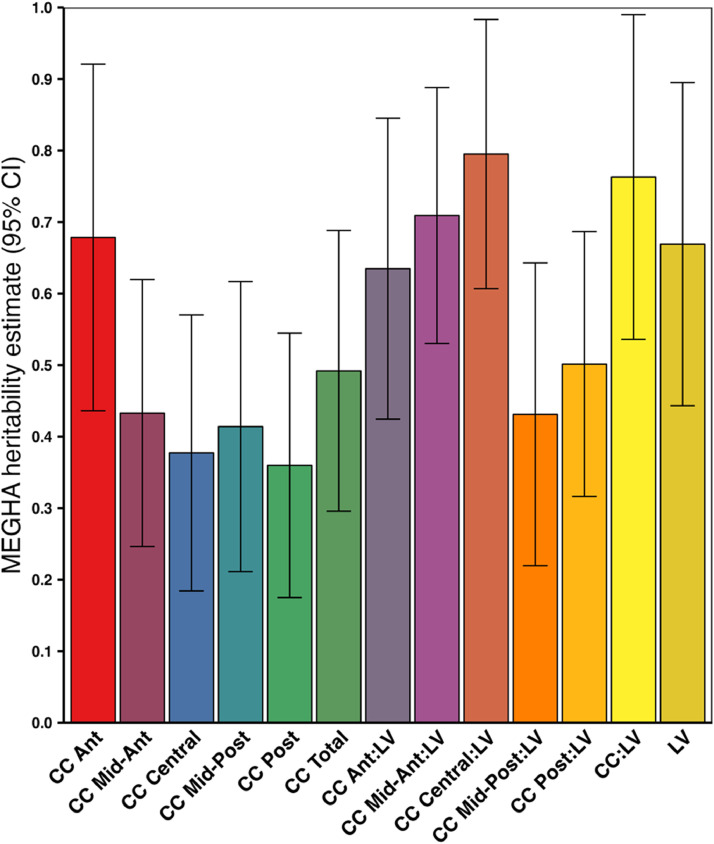
SNP-based heritability estimates for volumes of lateral ventricles and corpus callosum obtained using Massively Expedited Genome-wide Heritability Analysis (MEGHA). Abbreviations: Ant = Anterior, CC = corpus callosum, CI = confidence interval, LV = lateral ventricles, Post = Posterior, SNP = single nucleotide polymorphism. * *p* < 0.05, FDR-corrected.

MEGHA heritability estimates ranged from 0.36 for CC posterior up to 0.76 for the CC:LV ratio and 0.79 for the CC central:LV ratio (Fig. 3).

### Role of clinical and cognitive variables

**Fig. 4:**
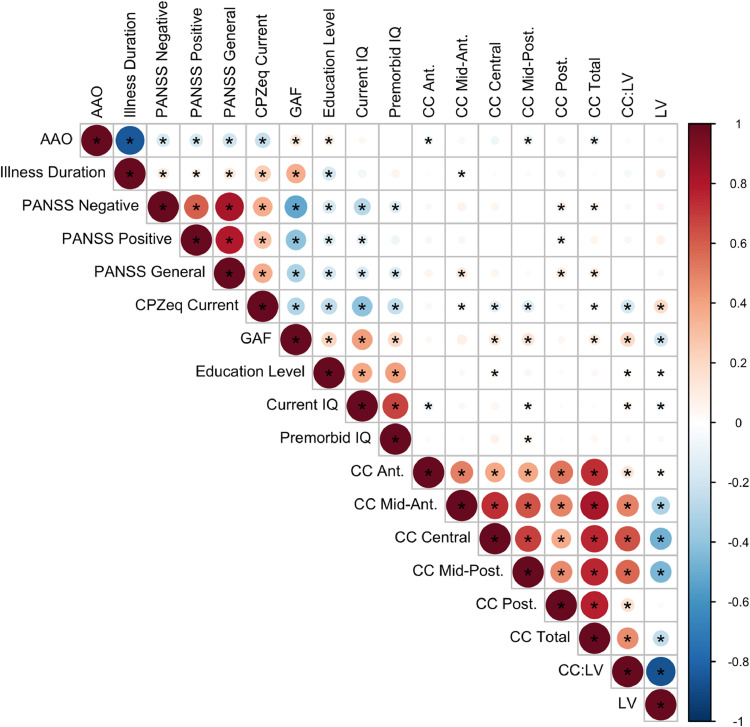
Correlations between volumes of lateral ventricles and corpus callosum, and clinical measures in patients and controls. * *p* < 0.05, FDR-corrected. Of the clinical/demographic measures only education level, GAF, and IQ were available in controls.

Spearman partial correlations between LV and CC and clinical/cognitive variables were estimated (Fig. 4). Correlations were high between neuroimaging phenotypes. Of the clinical variables, the GAF score significantly correlated with multiple CC subregions, LV and most strongly with CC:LV ratio (see Supplementary Figure 2 for correlations including the FHR group).

## Discussion

By examining genetic associations of MIR137-regulated pathways with LV and CC abnormalities in SZ, and the role of cognitive, symptom and functional measures in these brain phenotypes, within a large collection of SZ and HC individuals from the GENUS Consortium, we show that the ratio of CC:LV indexes psychopathology and functioning beyond the CC and LV measures considered separately. Polygenic score associations furthermore point to the ephrin pathway as an essential regulator of the white matter tracts comprising the CC. These results also indicate that different pathways regulated by MIR137 associate specifically to different portions of the CC and/or LV, or alternatively with LV/CC ratio. In validating the data, all phenotypes displayed the expected SZ/HC differences, with increased LV volumes (∼ 0.5 SD above controls), and decreased CC volumes in SZ (∼ 0.5 SD below controls). This is consistent with previous studies that report increased LV volumes (e.g., (del *Re* et al., 2019, 2016b; Heller et al., 2021)) and reduced callosal volumes in large samples of SZ patients (Francis et al., 2016; Patel et al., 2015). The CC:LV ratio was significantly and positively associated with the GAF in this case-control sample, thereby confirming our results in a small sample of probands (CIDAR) (del Re et al., 2016b).

Importantly, MIR137, an established susceptibility locus in schizophrenia GWAS (Schizophrenia Working Group of the Psychiatric Genomics Consortium, 2014; Trubetskoy et al., 2022), acts as a regulator of several genes that are themselves significantly associated with SZ (Collins et al., 2014; Cosgrove et al., 2017; Kwon et al., 2013). Our hypothesis was that MIR137 and its regulated pathways would be associated with LV, CC, and their ratio, and explain a significant portion of the variance in these regions. The findings largely confirm this hypothesis and indicate that CC (portion):LV ratios show some of the strongest associations with the pathway PRS, in addition to LV volume. Among the 5 pathway PRSs, including ephrin, LTP, PKA, axon guidance signaling, and the MIR137-target pathway (Hill et al., 2014), the ephrin pathway associated with the most phenotypes, with the highest percentage of variance explained in the LV volume and CC (portion):LV ratios. Novel SNP-based heritability estimates of the CC subregions and CC:LV ratios in particular, indicate high heritability of the endophenotypes, with the highest heritability for the CC:LV ratio. Ventriculomegaly as well as abnormalities of the CC are present in several neuropsychiatric disorders including Alzheimer's disease, dementia, SZ, bipolar disorder, major depression, as well as Parkinson's and Huntington's disease (Duy et al., 2022; Kelly et al., 2018; Maxfield et al., 2023; Thompson et al., 2020), suggest partially common mechanisms of these diseases. While in some instances enlarged LV might index neurodegenerative syndromes with loss of brain matter, in neurodevelopmental syndromes it might index atypical neurodevelopment.

Our overall findings are consistent with previous reports suggesting that MIR137-associated risk for SZ may relate to broader downstream genetic effects (Cosgrove et al., 2017) since MIR137 acts as a critical upstream regulator of neurodevelopmental genetic pathways that affect morphometric brain changes. In this context, the most notable result concerns the ephrin pathway as an essential regulator of the white matter tracts composing the CC. Ephrin receptor tyrosine kinases and their ligands, the ephrins, which are abundant in the developing brain, regulate developmental processes that are crucial for correct brain formation, including complex short-range cell-cell and long-range interactions. Gene mutations of the ephrin family are also implicated in neurodevelopmental disorders such as lissencephaly, polymicrogyria, or heterotopia, and ZIGA virus neurodevelopmental abnormalities, although the underlying molecular mechanisms remain to be elucidated (Gerstmann & Zimmer, 2018).

Molecularly engineered EphB1 and B2 receptors in mice (Robichaux et al., 2016), preferentially produce rostral, vs caudal or rostral plus caudal, partial agenesis of the CC, according to different genotypes, suggesting specific mechanisms of ephrins in the development of the anterior versus posterior portions of the CC. In post-mortem SZ patient tissues (Saia-Cereda et al., 2016), and in organoids obtained from SZ-derived IPCs cells (Nascimento et al., 2022), the ephrin-B is one of the top dysregulated pathways in SZ.

Other studies have focused on other brain regions in relation to MIR137 in SZ. Patel et al. (2015), for example, in a sample that partly overlaps with this study (MCIC, TCD, NUIG), found that the homozygous MIR137 risk genotype in SZ was associated with an attenuated reduction of mid-posterior CC volume, along with trend-level effects in the adjacent central and posterior CC, although this study did not investigate genetic pathways regulated by MIR137, such as ephrin.

Cosgrove et al. (2018), in a sample also included in this study (TCD), observed a nominally significant association between increasing MIR137 PRS and decreasing brain volume, independent of diagnosis status, although there was no significant association between MIR137 PRS and cortical thickness, surface area or hippocampal volume, yielding only suggestive evidence of MIR137 impact on cortical structure. On the other hand, other studies have shown a relation between MIR137 and hippocampal volumes (Lett et al., 2013). Cosgrove et al. (2017), in another sample included in this study (TCD, NUIG), found that increased polygenic risk (*p* < 0.05) within the empirically derived MIR137 regulated gene score associated with lower performance on working and episodic memory, and IQ.

In a further study of a sample that also partly overlaps with this study (MCIC), SZ patients homozygous for the MIR137 risk allele showed significant decreases in occipital, parietal and temporal lobe GM concentration (GMC), with increasing MIR137-regulated PRS, whereas those carrying the protective minor allele showed significant increases in GMC with PRS (Wright et al., 2016). No correlations of GMC and PRS were found in HC.

In a Chinese cohort, Kuswanto et al. (2015) found that patients diagnosed with SZ who carry the risk homozygous genotype for one of two intronic MIR137 risk variants had decreased FA (i.e., decreased brain WM integrity) in the fronto-striatal regions compared to heterozygous genotype carriers. They also had worse attention and processing speed, and worse negative symptoms compared with the non-risk allele. Finally, Cummings et al. (2013), in a sample included in this study (TCD, NUIG), found that carriers of the MIR137 rs1625579 risk allele had lower scores for a positive symptom factor derived from the Operational Criteria Checklist for Psychotic Illness and Affective Illness (OPCRIT+) and lower scores on a lifetime measure of psychosis incongruity.

## Limitations

This study has limitations associated with sample diversity. The PRS itself only captures a proportion of genetic risk, and the PGC datasets are largely based on European ancestry samples. Therefore, PRS may not be accurate for other populations in GENUS, which include ∼25 % non-European ancestry participants. However, our sensitivity analyses comparing the analysis of European individuals only with the analysis of European+East Asian individuals provided some evidence of cross-ancestry effects.

## Summary

Notwithstanding limitations, we observed that the CC:LV ratio positively correlates with global functioning, as found in our previous small sample (del Re et al., 2016b). It is also consistent with the central and supportive role that the CC and LV provide for the rest of the brain, i.e. serving as a conduit for inter-hemispheric information transmission, and helping keep the brain buoyant, cushioned, nourished, and cleared of waste, respectively. We also highlight the importance of specific MIR137-regulated pathways in the morphometry of both LV and CC; with specific effects of pathways for different portions of the CC. We add here measures of heritability for the regions of interest, calculated with MEGHA. These findings indicate high heritability of these variables, including the CC:LV ratio. A major strength of this study is its size. A further important strength is the heritability finding that suggest that the highly heritable CC:LV ratio, with variance explained by the MIR137-regulated ephrin pathway, is a biomarker of SZ that correlates with global functioning.

## Declaration of competing interest

The authors declare that they have no known competing financial interests or personal relationships that could have appeared to influence the work reported in this paper.

The author is an Editorial Board Member/Editor-in-Chief/Associate Editor/Guest Editor for *International Journal of Clinical and Health Psychology* and was not involved in the editorial review or the decision to publish this article.

## Appendix Supplementary materials

Image, application 1

Image, application 2
