# Supplementary material for: MIR137 polygenic risk for schizophrenia and ephrin-regulated pathway: Role in lateral ventricles and corpus callosum volume
Source: Int J Clin Health Psychol. 2024 Apr 9;24(2):100458. doi: 10.1016/j.ijchp.2024.100458 (PMC11017057; doi:10.1016/j.ijchp.2024.100458)
Supplement: Supplementary file 1 [file mmc1.docx]

**Supplementary Materials for:**

**MIR137 polygenic risk for schizophrenia**

**and ephrin-regulated pathway:**

**Role in lateral ventricles and corpus callosum volume**

**Table of Contents**

Supplementary Methods 2

Cohorts 2

Demographic Measures 2

Clinical Measures 2

Cognitive Measures 3

Supplementary Tables 4

Supplementary Table 1. Compositions of the risk scores. 4

Supplementary Table 2. Demographic/Clinical characteristics of the GENUS Consortium subset with MRI data ^a^, including familial high-risk (FHR). 5

Supplementary Table 3. Polygenic Risk Score (PRS) association results for MIR137 relevant pathways. 6

Supplementary Figures 7

Supplementary Figure 1. Means and distributions of volumes of lateral ventricles and corpus callosum in GENUS, including familial high-risk (FHR). 7

Supplementary Figure 2. MIR137 Pathway Polygenic Risk Score (PRS) associations for cases and controls combined, excluding familial high-risk (FHR) – European ancestry. 8

Supplementary Figure 3. Correlations between volumes of lateral ventricles and corpus callosum, and clinical measures, across diagnostic groups, including familial high-risk (FHR). 14

Acknowledgements and conflicts of interest 15

Supplementary References 19

# Supplementary Methods

### Cohorts

Detailed information about the cohorts included here can be found in the source papers cited below, and in the GENUS Consortium project description (Blokland et al., 2018). Cohorts included in this study are Center for Addiction and Mental Health (CAMH) (Nazeri et al., 2013; Wheeler et al., 2014); Boston CIDAR Study / Veterans Affairs Healthcare System (CIDAR/VA) (Clemm von Hohenberg et al., 2014; del Re et al., 2014; Kikinis et al., 2010; Kikinis et al., 2015; Pasternak et al., 2012; Quan et al., 2013; Seitz et al., 2016); Genetics and Psychosis study (GAP) (O'Connor et al., 2013; Reis Marques et al., 2014; Theleritis et al., 2014; Vassos et al., 2017); Institute of Mental Health, Singapore Imaging Genetics and Neuropsychological Research in Psychosis (IMH-SIGNRP) (Ho et al., 2016; Kuswanto et al., 2015); King’s College London - Maudsley Twin Study (KCL-MTS) (Owens et al., 2012; Owens, Picchioni, et al., 2011; Owens, Rijsdijk, et al., 2011; Psychosis Endophenotypes International Consortium et al., 2014; Toulopoulou et al., 2007); Language and Risk in Schizophrenia (L&R) (Francis et al., 2012); Mind Clinical Imaging Consortium (MCIC) (Gollub et al., 2013) Massachusetts General Hospital (MGH) (Ho et al., 2016; Holt et al., 2012; Linnman et al., 2013; Roffman et al., 2013; Roffman et al., 2011; Roffman et al., 2007; Roffman, Weiss, Deckersbach, et al., 2008; Roffman, Weiss, Purcell, et al., 2008); New England Family Study (NEFS) (Buka et al., 2013; Goldstein et al., 2010; Goldstein et al., 2014; Seidman et al., 2013; Seidman et al., 2002); Pittsburgh High Risk Study (PHRS) (Bhojraj et al., 2011; Bhojraj et al., 2009); Trinity College Dublin and National University of Ireland, Galway (TCD/NUIG) (Donohoe et al., 2009; O'Donovan et al., 2008; Walters et al., 2010; Walters et al., 2013); University Medical Centre Utrecht, Schizophrenia study 1 (UMCU-SZ1) (Hulshoff Pol et al., 2001; Terwisscha van Scheltinga et al., 2012; Terwisscha van Scheltinga, Bakker, van Haren, Derks, Buizer-Voskamp, Boos, et al., 2013; Terwisscha van Scheltinga, Bakker, van Haren, Derks, Buizer-Voskamp, Cahn, et al., 2013); and University Medical Centre Utrecht, Schizophrenia study 2 (UMCU-SZ2) (Boos et al., 2013; Korver et al., 2012; Rais et al., 2012; Terwisscha van Scheltinga, Bakker, van Haren, Derks, Buizer-Voskamp, Boos, et al., 2013).

### Demographic Measures

Education level in years was obtained for most cohorts (CIDAR/VA, GAP, IMH-SIGNRP, KCL-MTS, L&R, MCIC, MGH, PHRS, UMCU-SZ1). For cohorts that obtained categorical measures of education level (CAMH, NEFS, TCD/NUIG, UMCU-SZ2), these were converted to approximate education level in years, assuming a formal education starting age of 6 years, a primary/elementary school completion age of 12 (i.e., 6 years education), a high school completion age of 18 (i.e., 12 years education), and university/college completion age of 22 (i.e., 16 years education).

### Clinical Measures

Most patients had symptom rating scales available, either Positive and Negative Syndrome Scale (PANSS) (Kay et al., 1987) or Scale for the Assessment of Negative Symptoms and Scale for the Assessment of Positive Symptoms (SANS/SAPS) (Andreasen, 1983, 1984). SANS/SAPS scores were converted to PANSS scores using the method described by van Erp et al. (2014), and only harmonized PANSS scores were analyzed. The Global Assessment of Functioning (GAF) was used for examining functional outcome (American Psychiatric Association, 1994). Current antipsychotic medication dosages were converted to (100 mg) chlorpromazine equivalents based on published dosage equivalence estimates (Gardner et al., 2010; Woods, 2003). Additionally, age at onset and duration of illness in years were available.

### Cognitive Measures

Premorbid IQ was estimated from reading tests, namely Wechsler Test for Adult Reading (Wechsler, 2001); Wide Range Achievement Test, Third or Fourth Edition (Wilkinson, 1993; Wilkinson & Robertson, 2006); or National Adult Reading Test (Nelson, 1982), or from the Wechsler Adult Intelligence Scale Vocabulary subtest (WAIS; (Wechsler, 1999)) if reading tests were not available. Current IQ was estimated based on one to eight WAIS subtests (Wechsler, 1974, 1981, 1997, 1999, 2005); see Blokland et al. (2018) for details.

# Supplementary Tables

### Supplementary Table 1. Compositions of the risk scores.

|  | N genes | N variants p<=1 | N variants p<0.5 | N variants p<0.05 | N variants p<1e-5 | N variants p<5e-8 |
| --- | --- | --- | --- | --- | --- | --- |
| MIR137-gene | 1 | 129 | 122 | 107 | 45 | 42 |
| MIR137-EphR | 25 | 11298 | 7245 | 2296 | 156 | 10 |
| MIR137-LTP | 13 | 9059 | 5357 | 1534 | 172 | 147 |
| MIR137-PKA | 45 | 45521 | 26758 | 5163 | 190 | 88 |
| MIR137-AxG | 42 | 21924 | 12875 | 3125 | 8 | 0 |
| MIR137 targets (Hill et al., 2014) | 831 | 371010 | 226719 | 65734 | 11114 | 7236 |
| PGC-SZ2 | 25888 | 9444230 | 5422836 | 1084993 | 34628 | 12897 |

EphR = Ephrin receptor signaling; LTP = synaptic long-term potentiation; PKA = Protein kinase cAMP-dependent signaling; AxG = axon guidance signaling; PGC-SZ2 = Psychiatric Genomics Consortium SZ PRS

N genes is based on ucsc b37/refGene_gene_positions and autosomal genes only.

### Supplementary Table 2. Demographic/Clinical characteristics of the GENUS Consortium subset with MRI data ^a^, including familial high-risk (FHR).

|  | **Patients** | | **Controls** | | **FHR** | | **Statistic (F)** | **df** | **p** | **Posthoc comparisons adjusted p-values ^e^** | | |
| --- | --- | --- | --- | --- | --- | --- | --- | --- | --- | --- | --- | --- |
|  | **N** | **Mean ± SD (Range)** | **N** | **Mean ± SD (Range)** | **N** | **Mean ± SD (Range)** |  |  |  | **PT-HC** | **FHR-HC** | **PT-FHR** |
| **Age (years)** | 1224 | 34.5±11.7  (14–76) | 1466 | 34.8±12.6  (11–86) | 378 | 27.1±9 (  10.1–65) | 68.3 | 2, 3065 | <0.001 | 0.73 | <0.001 | <0.001 |
| **Education Level (years) ^b^** | 1105 | 12.3±2.7  (3–22) | 1136 | 14.2±2.7  (4–26) | 374 | 12.8±3.2  (3–20) | 130.5 | 2, 2612 | <0.001 | <0.001 | <0.001 | 0.02 |
| **Premorbid IQ** | 586 | 101.1±14.5  (56–145) | 598 | 109.8±11.2  (67–138) | 44 | 111±11.9  (82–134) | 70.4 | 2, 1225 | <0.001 | <0.001 | 0.82 | <0.001 |
| **Current IQ** | 601 | 97.3±17  (51–155) | 627 | 114.6±14.4  (72–155) | 276 | 104.2±15  (70–152) | 189.8 | 2, 1501 | <0.001 | <0.001 | <0.001 | <0.001 |
| **Age at Onset (years)** | 1036 | 23±7.5  (3–58) | --- | --- | --- | --- | --- | --- | --- | --- | --- | --- |
| **Illness Duration (years)** | 1072 | 11.3±11.5  (0–53) | --- | --- | --- | --- | --- | --- | --- | --- | --- | --- |
| **PANSS Positive ^c^** | 858 | 14.3±5.9  (7–41) | --- | --- | --- | --- | --- | --- | --- | --- | --- | --- |
| **PANSS Negative ^c^** | 855 | 13.9±6.1  (7–42) | --- | --- | --- | --- | --- | --- | --- | --- | --- | --- |
| **PANSS General** | 754 | 28.7±11.3  (0–93) | --- | --- | --- | --- | --- | --- | --- | --- | --- | --- |
| **Global Assessment of Functioning** | 342 | 54.2±16.7  (11–100) | --- | --- | --- | --- | --- | --- | --- | --- | --- | --- |
| **Chlorpromazine equivalent ^d^ current antipsychotic dose (mg)** | 748 | 316±339.6  (0–2900.2) | --- | --- | --- | --- | --- | --- | --- | --- | --- | --- |
|  | **N** | **%** | **N** | **%** | **N** | **%** | **Statistic (χ^2^)** | **df** | **p** |  |  |  |
| **Sex (female / male)** | 373 / 852 | 30.1 / 69.9 | 700 / 766 | 47.7 / 52.3 | 210 / 168 | 55.6 / 44.4 | 116.2 | 2 | <0.001 | --- | --- | --- |
| **Ancestral Population (EUR / EAS / AFR / AMR / SAS / MIX)** | 571 / 149 / 102 / 23 / 16 / 9 | 65.6 / 17.1 / 11.7 / 2.6 / 1.8 / 1.0 | 891 / 21 / 83 / 29 / 19 / 0 | 85.4 / 2.0 / 8.0 / 2.8 / 1.8 / 0 | 214 / 1 / 30 / 2 / 3 / 0 | 85.6 / 0.4 / 12.0 / 0.8 / 1.2 / 0 | 208.7 | 10 | <0.001 | --- | --- | --- |
| **Medication status (medicated / unmedicated / unknown)** | 810 / 231 / 183 | 66.2 / 18.9 / 15.0 | --- | --- | --- | --- | --- | --- | --- | --- | --- | --- |

Abbreviations: AFR = African; AMR = American; EAS = East Asian; EUR = European; MIX = Mixed Ancestry; SAS = South Asian; IQ = Intelligence quotient; PANSS = Positive and Negative Syndrome Scale; PT = Patients; HC = Healthy Controls; FHR = Familial High-Risk; SD = Standard Deviation.

^a^ All available MRI data were used for standardization and covariate adjustment, regardless of availability of genetic data.

^b^ Education level is measured in years from age 6, i.e., 12 years of education indicates high school completion (in most countries).

^c^ Composite of PANSS and SANS/SAPS, calculated according to van Erp et al. (2014), is reported instead of scores from these scales separately, to increase sample size and reduce the multiple testing burden; ^d^ Antipsychotic dose equivalent to 100 mg chlorpromazine; ^e^ Tukey Posthoc comparisons adjusted p-values.

### Supplementary Table 3. Polygenic Risk Score (PRS) association results for MIR137 relevant pathways.

See SupplementaryTable3_PRS_LV_CC_metafor_all_results.xlsx

# Supplementary Figures


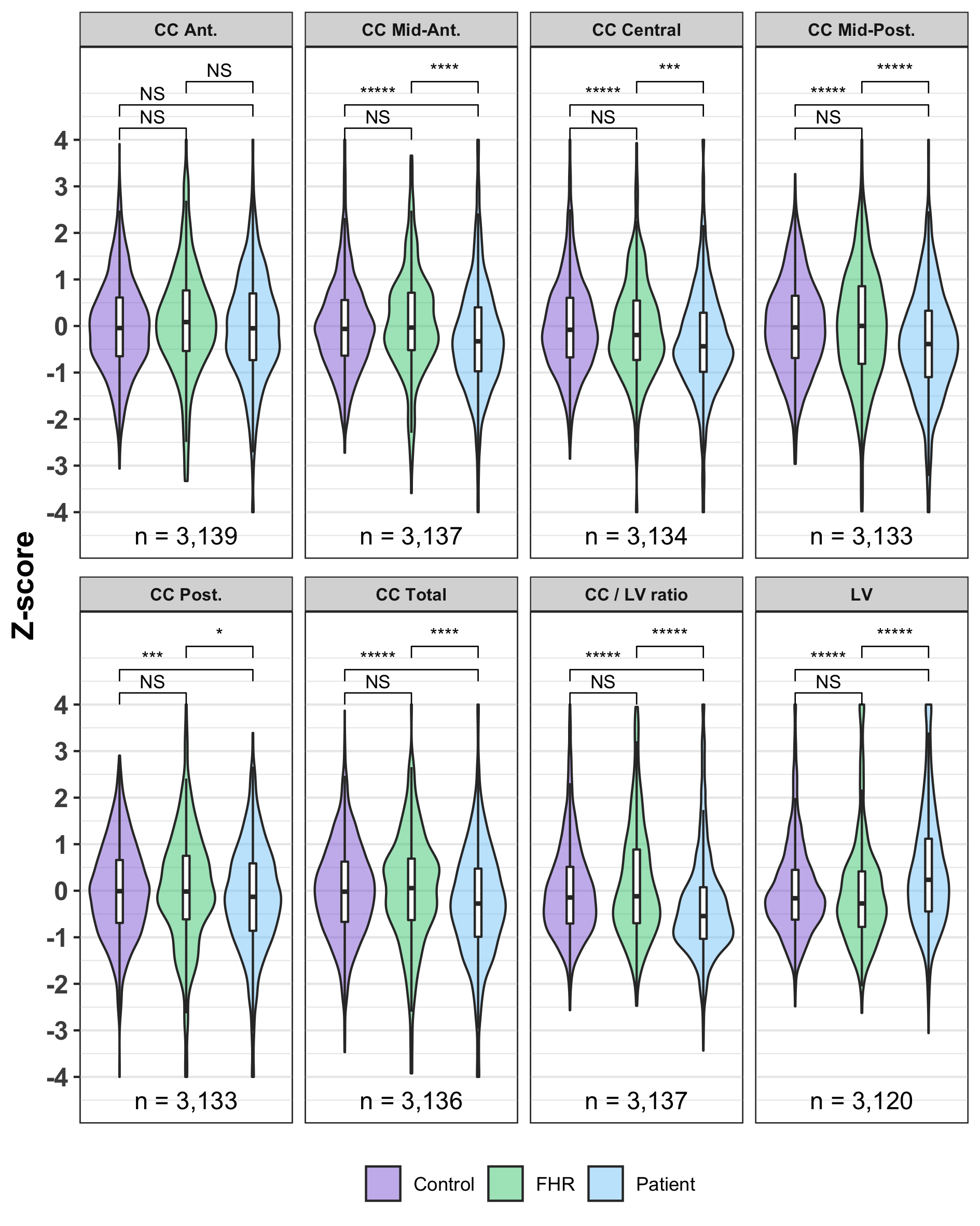


### Supplementary Figure 1. Means and distributions of volumes of lateral ventricles and corpus callosum in GENUS, including familial high-risk (FHR).

* p < 0.05; ** p < 0.01; *** p < 0.001; **** p < 0.0001; ***** p < 0.00001

Abbreviations: Ant. = Anterior, CC = corpus callosum, FHR = familial high-risk, LV = lateral ventricles, Post. = Posterior

### Supplementary Figure 2. MIR137 Pathway Polygenic Risk Score (PRS) associations for cases and controls combined, excluding familial high-risk (FHR) – European ancestry.

* p < 0.05, FDR-corrected for 13 phenotypes and 7 PRSs.

**a) Axon guidance pathway**

**
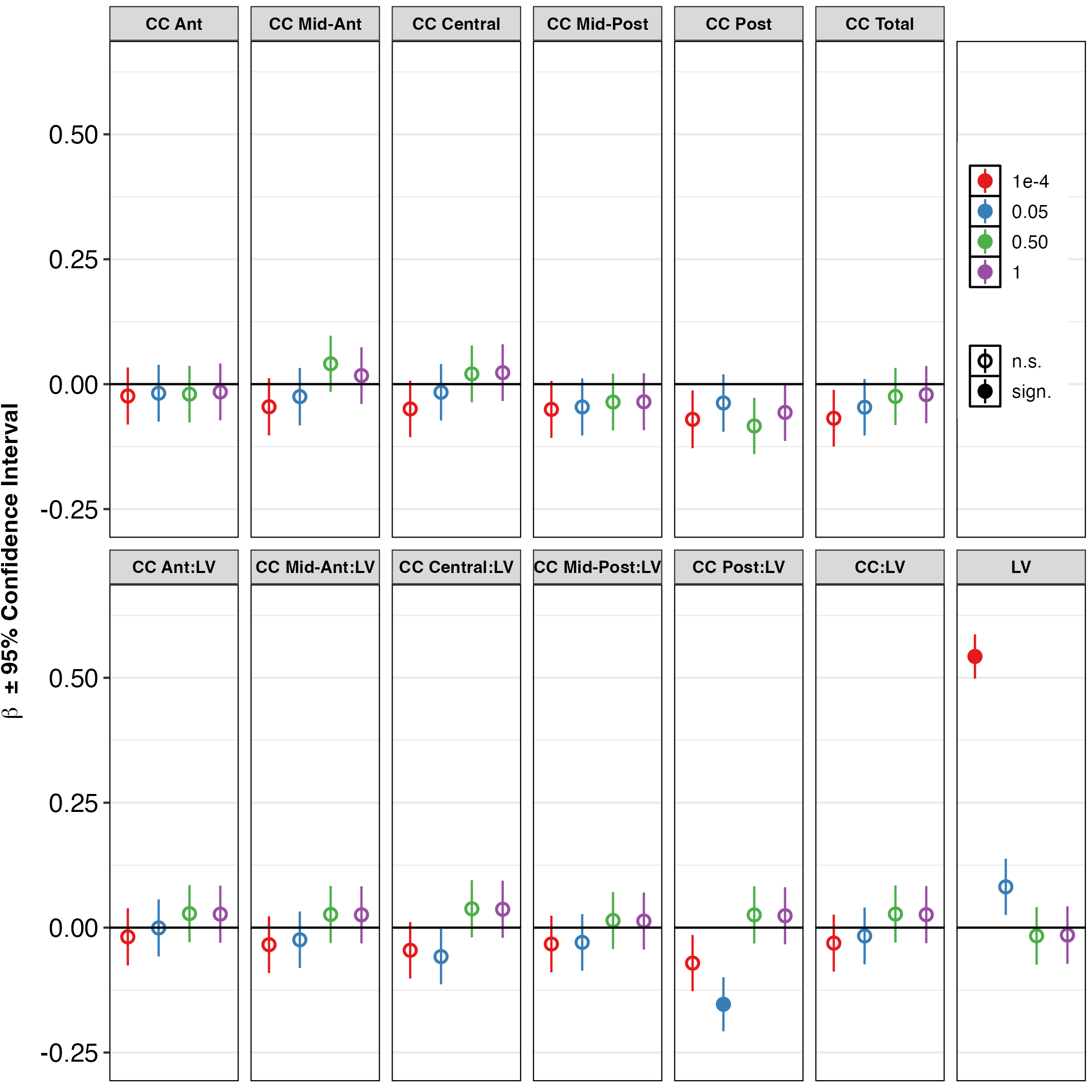
**

**b) Ephrin pathway**

**
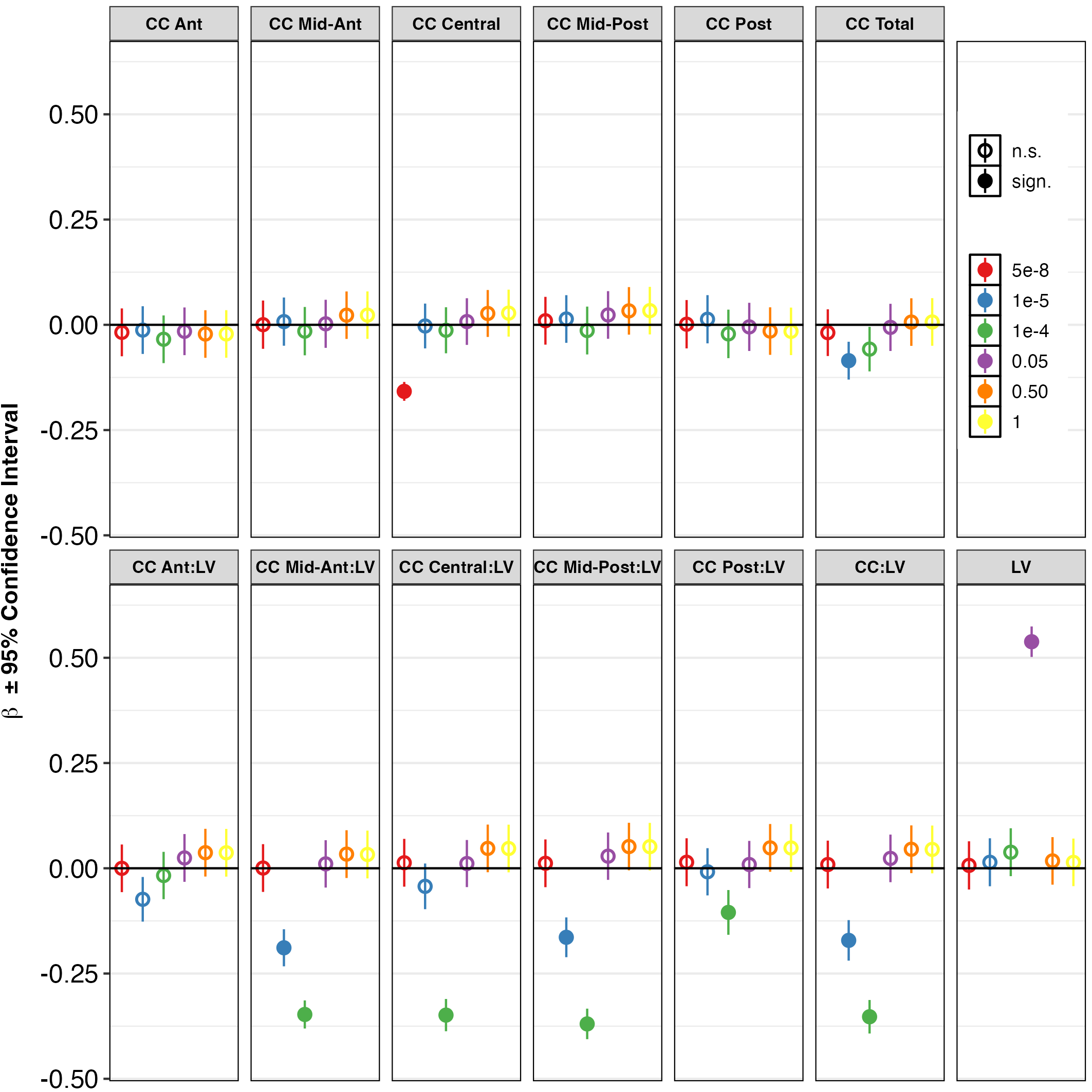
**

**c) Long-term potentiation pathway**

**
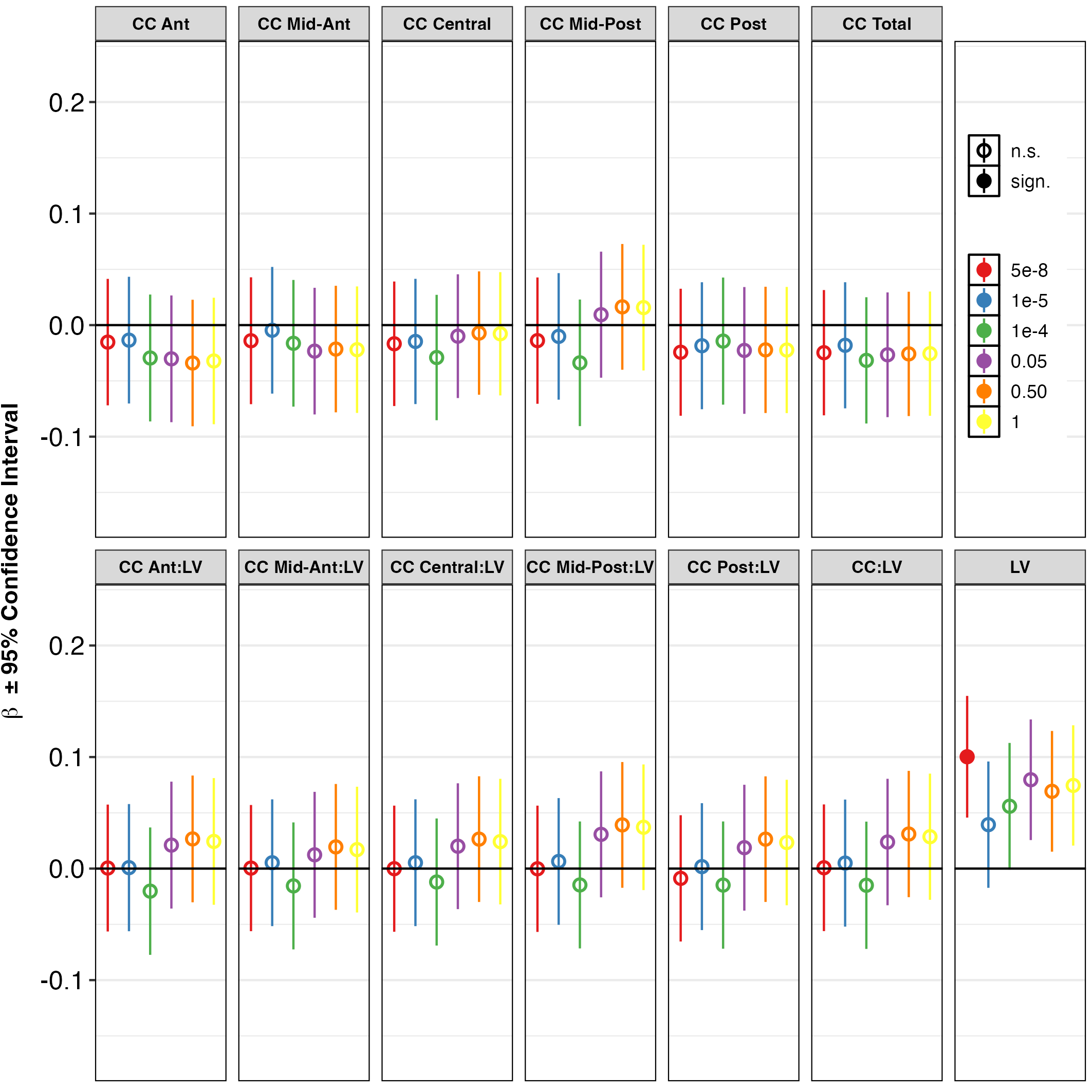
**

**d) PKA pathway**

**
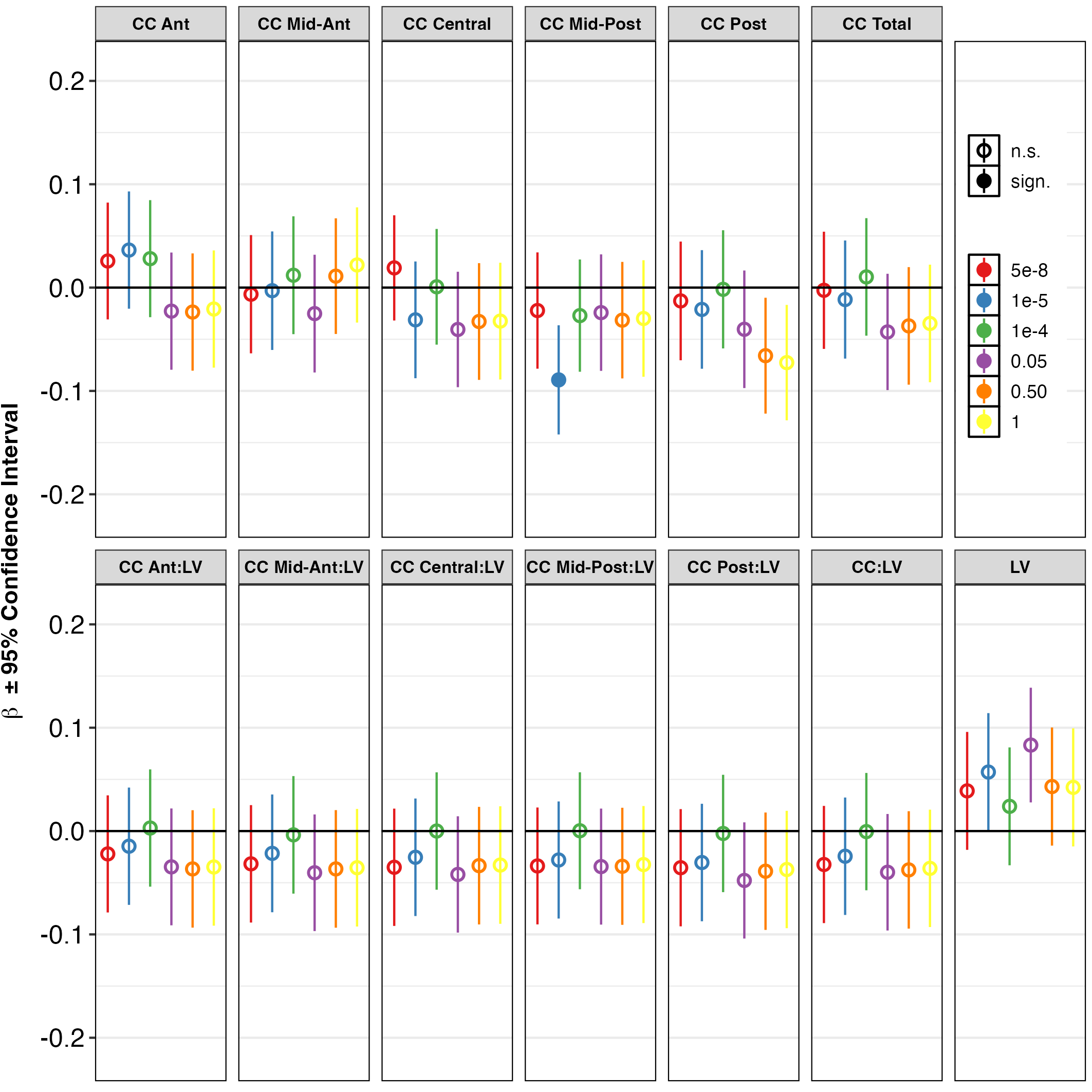
**

**e) MIR137 targets (Hill et al., 2014)**

**
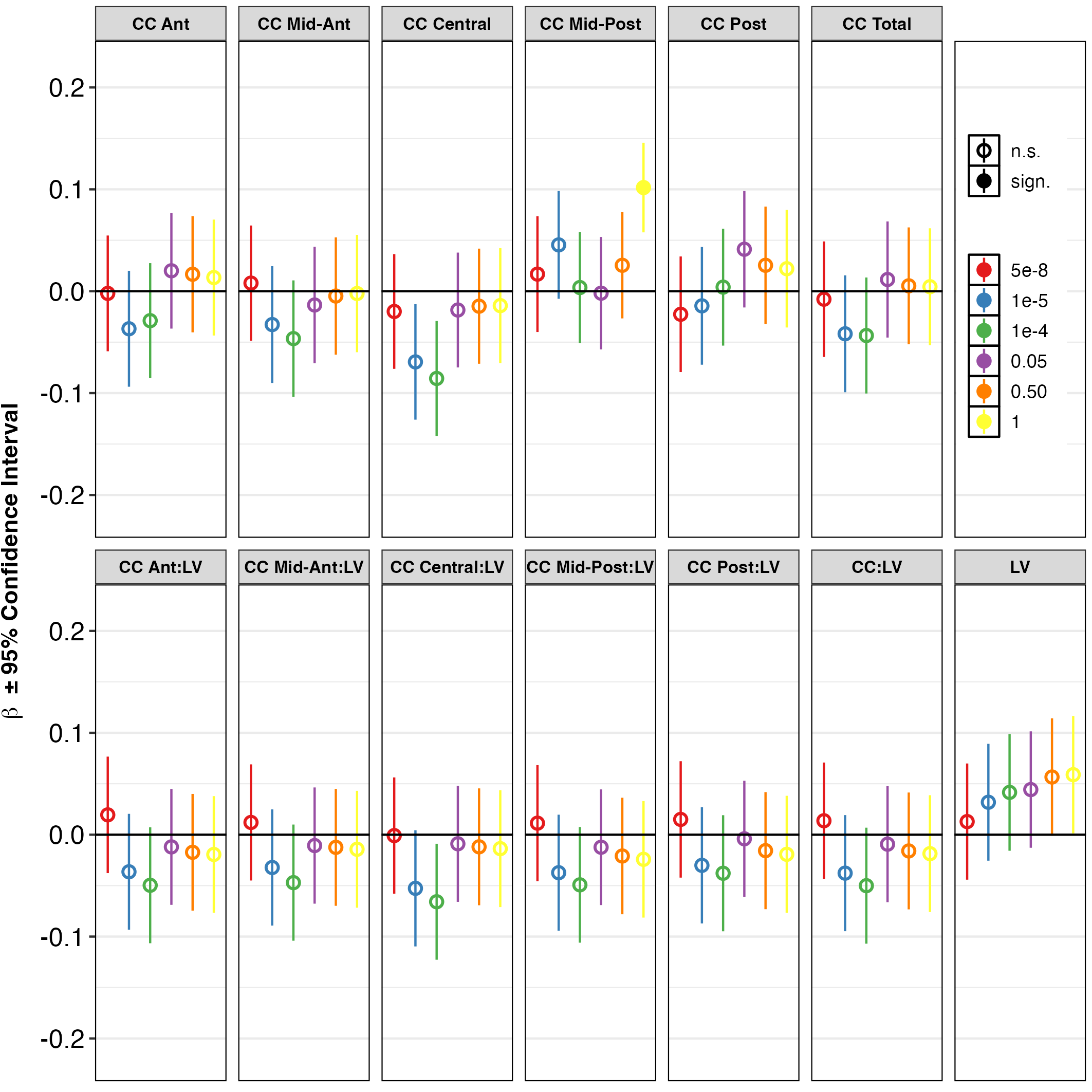
**

**f) MIR137 gene**

**g) PGC-SZ2 PRS (for comparison)**


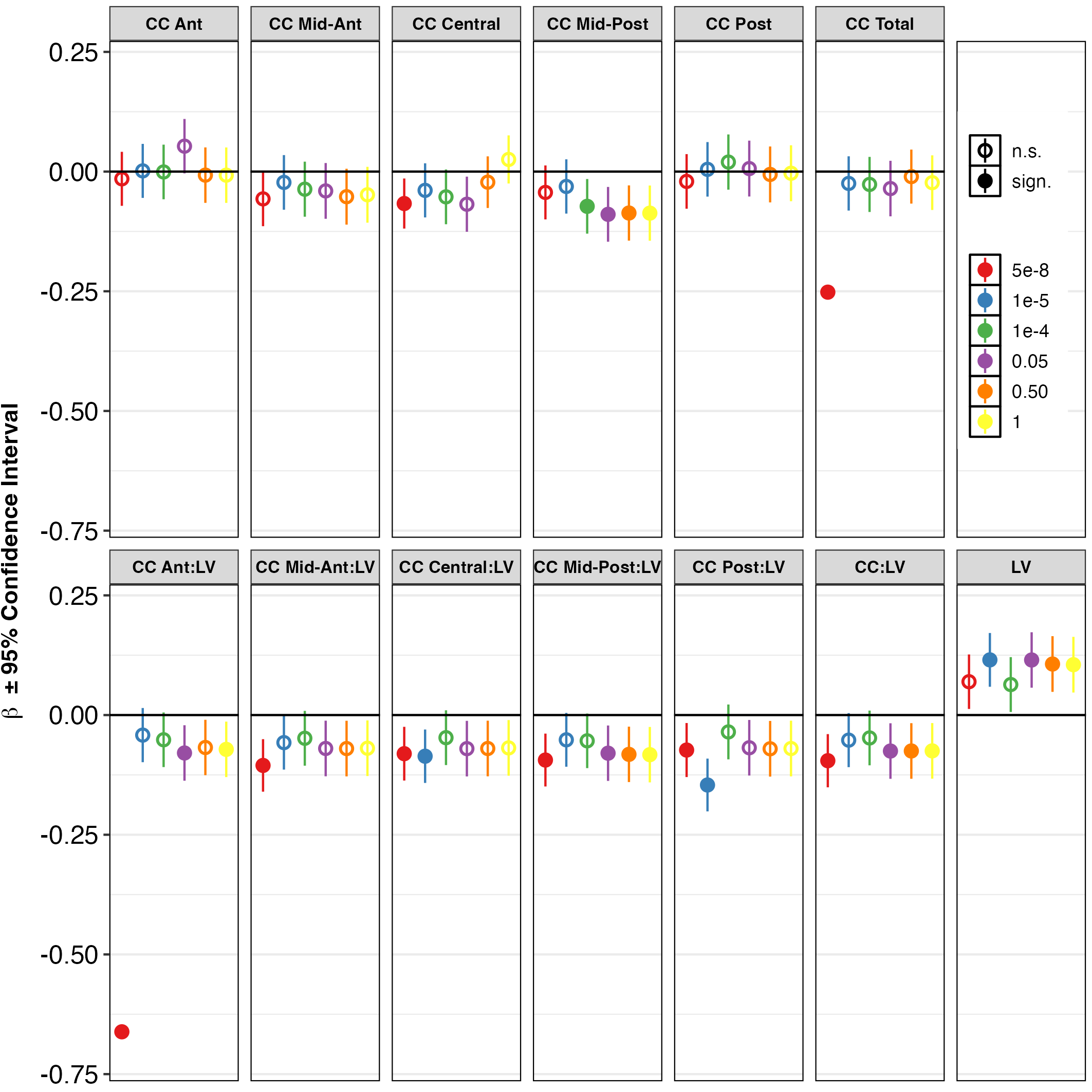


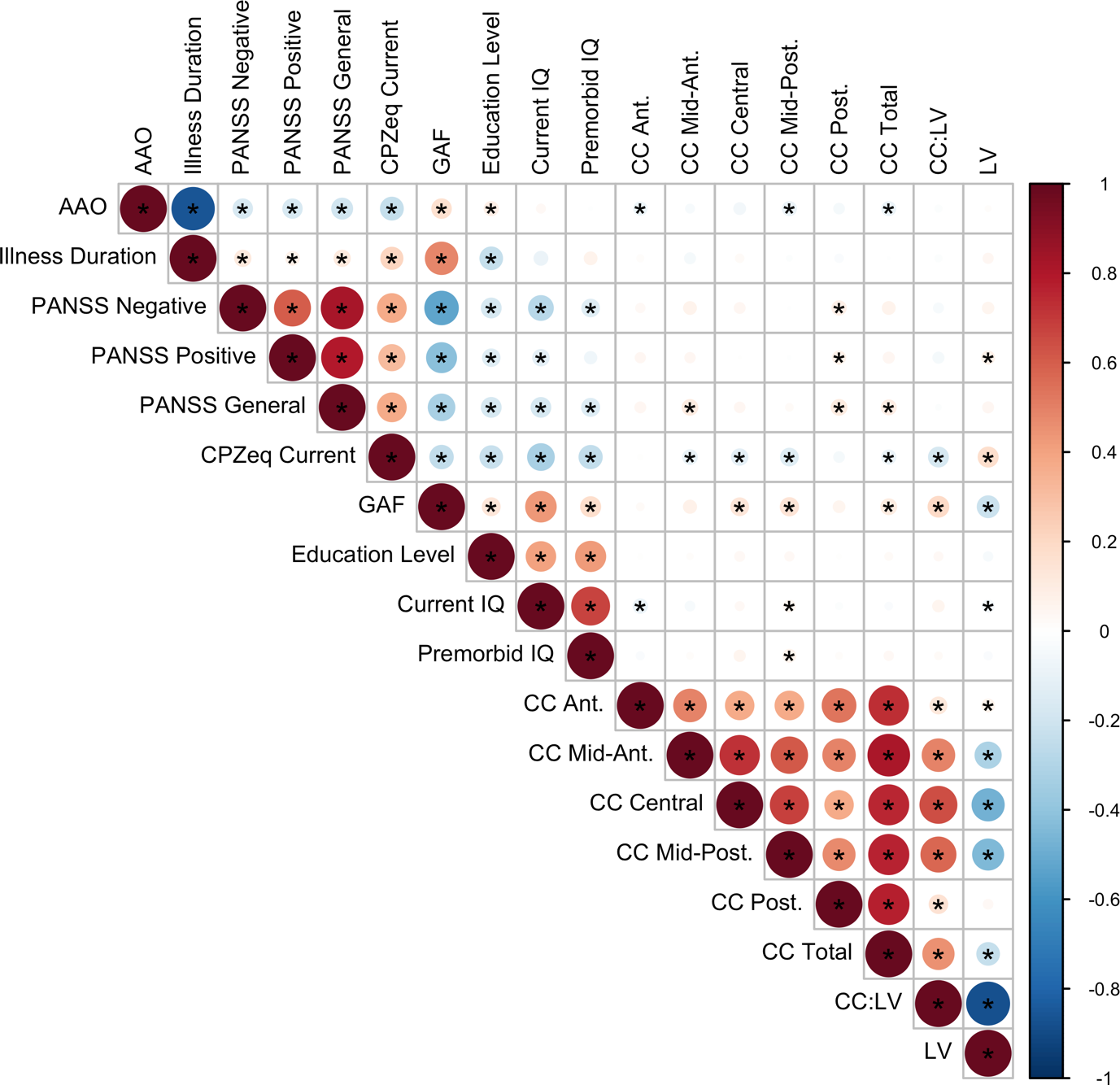


### Supplementary Figure 3. Correlations between volumes of lateral ventricles and corpus callosum, and clinical measures, across diagnostic groups, including familial high-risk (FHR).

* p < 0.05, FDR-corrected.

Of the clinical/demographic measures only education level, GAF, and IQ were available in controls.

Abbreviations: AAO = age at onset, Ant. = Anterior, CC = corpus callosum, CPZeq = Chlorpromazine equivalent dose (100 mg), GAF = Global Assessment of Functioning, IQ = intelligence quotient, LV = lateral ventricles, PANSS = Positive and Negative Syndrome Scale, Post. = Posterior

Composites of PANSS and SANS/SAPS, calculated according to van Erp et al. (2014), are reported instead of scores from these scales separately, to increase sample size and reduce the multiple testing burden.

# Acknowledgements and conflicts of interest (for legacy cohorts)—Please note that for submission (manuscript file is required to be anonymous) we are removing this portion for now, but it will be re-added in case the manuscript is accepted.

**CAMH — Center for Addiction and Mental Health**

Data acquisition was supported by the Canadian Institutes of Health Research, Ontario Mental Health Foundation, the Brain and Behavior Research Foundation (formerly NARSAD), the Centre for Addiction and Mental Health (CAMH), and the CAMH Foundation through the Kimel Family, Koerner New Scientist Award, and Paul E Garfinkel New Investigator Catalyst Award. The investigators acknowledge the assistance of David J. Rotenberg and Dr. Daniel Felsky.

**CIDAR/VA — Boston CIDAR Study / Veterans Affairs Healthcare System**

Data acquisition was supported by the National Institute of Mental Health (NIMH) of the National Institutes of Health (NIH) under Award Numbers P50 MH080272 (RW McCarley), U01MH081928 (LJ Seidman), R21MH106793 (Z Kikinis), and a NARSAD Young Investigator Award 2008 (Z Kikinis). Genotyping was funded by a Massachusetts General Hospital Executive Committee on Research Interim Support Fund award (TL Petryshen). The investigators thank all subjects for their participation in the study, the clinical and data management staff from the Boston CIDAR study and the Commonwealth Research Center (Dr. Matcheri Keshavan, Dr. Joanne Wojcik, Ann Cousins, Dr. Michelle Friedman-Yakoobian, Dr. Anthony J Giuliano, Andrea Gnong Granato, Lauren Gibson, Sarah Hornbach, Julia Schutt, Dr. Kristy Klein, Dr. Maria Hiraldo, Dr. Grace Francis, Corin Pilo, Rachael Serur, Grace Min, Alison Thomas, and Molly Franz), Tamara Tasoff, Dr. Beril Yaffe, and Danbee Kim for electrophysiology assistance (Harvard), and Kimberly Chambert (Broad Institute of MIT and Harvard), Patience Gallagher, Dr. Stephen Haddad, Brian Galloway, and Jenna Tarasoff (Massachusetts General Hospital) for genotyping assistance.

**GAP — Genetics and Psychosis study**

The GAP study was supported by the National Institute for Health Research (NIHR) Mental Health Biomedical Research Centre at South London and Maudsley NHS Foundation Trust and King’s College London. Dr. Dazzan’s research is also supported by the Medical Research Foundation. Dr. Reis-Marques and Dr. Dazzan’s research is supported by NARSAD. Dr. Reinders is supported by the Netherlands Organization for Scientific Research (NWO-VENI grant no. 451-07-009). Genotyping and basic QC was supported by Guy**’**s and St. Thomas Charity Grant No.R080529 (E Vassos) and the Psychiatry Research Trust (R Murray). The views expressed are those of the authors and not necessarily those of the NHS, the NIHR, or the Department of Health". The investigators are grateful for the support of all GAP researchers and Principal Investigators for their contribution and support of the GAP study, and gratefully acknowledge the help of the Genetic & Psychosis and Physical Health & Substance Use in First Episode Psychosis study teams and participants, South London & Maudsley Mental Health NHS Trust. The investigators wish to specifically acknowledge Dr. Carmine M. Pariante, Dr. Kie Woo Nam, and Heather Taylor.

*Conflicts of interest.* Dr. Robin Murray has received honoraria for lectures from Janssen, AstraZeneca, Lilly, Novartis and Bristol-Myers Squibb.

**IMH-SIGNRP — Institute of Mental Health, Singapore Imaging Genetics and Neuropsychological Research in Psychosis**

Data acquisition was supported by the National Research Foundation Singapore under the National Medical Research Council Translational and Clinical Research Flagship Programme (grant no. NMRC/TCR/003/2008) for The Singapore Translational and Clinical Research in Psychosis, as well as research grants from the National Medical Research Council under the Centre Grant Programme (Institute of Mental Health, Singapore) (NMRC/CG/004/2013), the National Healthcare Group, Singapore (SIG/05004; SIG/05028), and the Singapore Bioimaging Consortium (RP C-009/2006). The investigators wish to acknowledge the contribution of Mingyuan Wang.

**KCL-MTS — King’s College London - Maudsley Twin Study**

Support was provided by the European Community’s Sixth Framework Programme through a Marie Curie Training Network (MRTN-CT-2006-035987) called the European Twin Study Network on Schizophrenia (EUTwinsS), NARSAD (through a Young Investigator Award to Dr. Toulopoulou), Wellcome Trust Research Training Fellowship (grant 064971 to Dr. Picchioni), Economic and Social Research Council/Medical Research Council and the Psychiatry Research Trust (PTA-037-27-0002). The investigators acknowledge support from the Department of Health via the National Institute for Health Research (NIHR) Specialist Biomedical Research Centre for Mental Health award to South London and Maudsley NHS Foundation Trust (SLaM) and the Institute of Psychiatry at King’s College London. Principal funding for genotyping was provided by the Wellcome Trust, as part of the Wellcome Trust Case Control Consortium 2 project (Grant Nos. 085475/B/08/Z and 085475/Z/08/Z). Dr. Bramon was supported by a MRC New Investigator Award, a MRC Centenary Award, the National Institute of Health Research UK (post-doctoral fellowship), the Psychiatry Research Trust, the Schizophrenia Research Fund, a Brain and Behavior Research Foundation (NARSAD) Young Investigator Award, a Wellcome Trust Research Training Fellowship, and the NIHR Biomedical Research Centre for Mental Health at the South London and Maudsley NHS Foundation Trust and Institute of Psychiatry Kings College London.

*Conflicts of interest.* Dr. Robin Murray has received honoraria for lectures from Janssen, AstraZeneca, Lilly, Novartis and Bristol-Myers Squibb. Dr. Picchioni has received travel awards from Pfizer, Janssen-Cilag, and Eli Lily and an educational grant from Janssen-Cilag.

**L&R — Language and Risk in Schizophrenia**

Data acquisition was funded by NIMH grant R21MH083205 (LE DeLisi), R01MH064023 (MS Keshavan), and the Commonwealth Research Center of the Massachusetts Department of Mental Health grant SCDMH82101008006 (LJ Seidman).

**MCIC — Mind Clinical Imaging Consortium**

The Mind Clinical Imaging Consortium was supported primarily by the Department of Energy DE-FG02-99ER62764 through its support of the Mind Research Network (MRN, formerly known as the MIND Institute) and the consortium, as well as by a NARSAD Young Investigator Award (to Dr. Ehrlich), the Blowitz-Ridgeway and Essel Foundations, NWO ZonMw TOP 91211021, DFG research fellowship (to Dr. Ehrlich), UK Research and Innovation (UKRI) under the UK government’s Horizon Europe / ERC Frontier Research Guarantee [BrainHealth, grant number EP/Y015037/1] (to Dr. Walton), the Mind Research Network, National Institutes of Health through NCRR 5MO1-RR001066 (MGH General Clinical Research Center), NIMH K08 MH068540, the Biomedical Informatics Research Network with NCRR Supplements to P41 RR14075 (MGH), M01 RR 01066 (MGH), NIBIB R01EB006841 (MRN), R01EB005846 (MRN), 2R01 EB000840 (MRN), 1RC1MH089257 (MRN), as well as grant U24 RR021992, P20RR021938/P20GM103472 and R01MH094524. The investigators wish to thank their many colleagues who served as mentors, advisors and supporters during the inception and conduct of the study including Donald Goff, Gina Kuperberg, Jill Goldstein, Martha Shenton, Robert McCarley, Stephan Heckers, Cynthia Wible, Raquelle Mesholam-Gately, and Mark Vangel.  The investigators thank the study staff and clinicians at each site that were responsible for the data acquisition, including Stuart Wallace, Ann Cousins, Raquelle Mesholam-Gately, Steven Stufflebeam, Oliver Freudenreich, Daphne Holt, Joshua Roffman, Laura Kunkel, Frank Fleming, George He, Hans Johnson, Ron Pierson, Arvind Caprihan, Phyllis Somers, Christine Portal, Kaila Norman, Diana South, Michael Doty and Haley Milner.  The investigators also acknowledge the expert guidance on image and other types of data acquisition obtained from Lee Friedman, Stephan Posse, Jorge Jovicich, and Tom Wassink, and acknowledge the many research assistants, students and colleagues who assisted in data curation over the years since data acquisition was completed, including Stuart Wallace, Carolyn Zyloney, Komal Sawlani, Esther Walton, Jill Fries, Adam Scott, Dylan Wood, Runtang Wang, William Courtney, Angie Guimaraes, Lisa Shenkman, Mustafa Kendi, Aysa Tuba Karagulle Kendi, Ryan Muetzel, Tara Biehl, and Marcus Schmidt.

**MGH — Massachusetts General Hospital**

The collection of the cognitive and clinical subsample was supported by NIH grants MH02025-01A3, MH60450 (Dr. Goff) and DK56085 (Dr. Halsted), as well as by a NARSAD Independent Investigator Award and the Sidney R. Baer Jr. Foundation (Dr. Goff), and an APIRE/Lilly Psychiatric Research Fellowship and Harvard Medical School Dupont-Warren Fellowship (Dr. Roffman). The acquisition of neuroimaging data for subsample 1 was funded by National Institute of Mental Health grants R01MH067720 (Dr. Manoach), R01MH070831 (Dr Goff), R01MH101425 and K23MH084059 (Dr. Roffman), a grant from Pamlab (Dr. Roffman), and the Howard Hughes Medical Institute Physician-Scientist Early Career Award (Dr Roffman). This work was conducted with support from Harvard Catalyst, The Harvard Clinical and Translational Science Center (National Center for Research Resources and the National Center for Advancing Translational Sciences, National Institutes of Health award UL1 RR 025758, and financial contributions from Harvard University and its affiliated academic health care centers). The acquisition of neuroimaging data for subsample 2 was supported by National Institute of Mental Health grants K23MH076054 and RO1MH095904 (Dr. Holt) and NARSAD with the Sidney R. Baer, Jr Foundation (Dr. Holt). The investigators thank David C. Henderson, Alexandra S. Tanner, Madeline Giegold, and Noah J. Silverstein, as well as Garth Coombs III, Adam Z. Nitenson, and Liana J. Petruzzi for their indispensable help with the MRI and clinical data acquisition, and Stephanie N. DeCross and Franklin C. Huntington for administrative support.

**NEFS — New England Family Study**

Original sample ascertainment was supported by NIMH R01MH50647 (1999–2003, Tsuang, P.I.; 2003–2006, Goldstein, P.I.), RO1 MH56956 (Goldstein, P.I.), and the Stanley Medical Research Institute (Buka and Goldstein). The authors would like to express their appreciation to the dedicated NEFS research team including Jasmina Burdzovic-Andreas, Ph.D., Monica Landi, M.S.W., June Wolf, Ph.D., and JoAnn Donatelli, Ph.D., for their contributions to the recruitment of subjects, clinical interviewing and diagnoses, Christiana Provencal, M.A., for her contribution to project management during the original sample acquisition. We also appreciate the efforts of a number of research assistants over the years and a previous project coordinator, Lisa Denny, MD, for their contributions to the study at an earlier stage of the work and Ronald O. Rieder, MD, for access to his earlier 1975 study of some of the Boston mothers in our parental case group. We would also like to thank Harlyn Aizley, Ed.M., Jenn Walch, M.Ed., for their immense contributions to the collection and management of data, respectively. The investigators furthermore wish to acknowledge Anne Remington, Lynda Tucker, and Dr. George Papadimitriou for their contributions.

**PHRS — Pittsburgh High Risk Study**

The study was funded by NIMH grant MH64023 (Dr. Keshavan). The investigators acknowledge the contributions of Dr. Vishwajit L. Nimgaonkar, Dr. Kodavali Chowdari, Dr. Debra M. Mostrose, Jean M. Miewald, Ian Mathew, Brandon M. Hager, and Olivia Lutz.

**TCD/NUIG — Trinity College Dublin and National University of Ireland, Galway**

Recruitment and genotyping were supported by the Wellcome Trust and Science Foundation Ireland (SFI08/IN.1/B1916, A Corvin). Dr Donohoe is also supported by the European Research Council (ERC-2015-STG-677467). The investigators thank Prof. John Waddington, Prof. Ted Dinan, Prof. Eadbhard O**’**Callaghan, Prof. Kieran Murphy, and Dr. F. Anthony O**’**Neil for their role in the recruitment of the patient sample.

**UMCU-SZ1 — University Medical Centre Utrecht, Schizophrenia study 1**

The study was supported by grants 7F99.(2).37 (H Hulshoff Pol) and 14F06(2)-34 (SC Bakker) from the HersenStichting Nederland (Dutch Brain Foundation), The Hague, the Netherlands, by a Veni grant from Zorg Onderzoek Nederland, Medische Wetenschappen to SC Bakker (the Dutch organization for health research and development, project number: 91686137), and by Top Institute Pharma (project T5-203). Genome-wide SNP genotyping was funded by NIMH grant R01MH078075 (RA Ophoff).

**UMCU-SZ2 — University Medical Centre Utrecht, Schizophrenia study 2**

The study was funded through the Geestkracht programme of the Dutch Health Research Council (ZON-MW, grant number 10-000-1001), and matching funds from participating industry (Lundbeck, AstraZeneca, Eli Lilly, Janssen Cilag), universities and mental health care organizations in the Netherlands. Genome-wide SNP genotyping was funded by NIMH grant R01MH078075 (RA Ophoff).

# Supplementary References

American Psychiatric Association. (1994). *Diagnostic and Statistical Manual of Mental Disorders, Fourth Edition (DSM-IV)*. American Psychiatric Press Inc.

Andreasen, N. C. (1983). *Scale for the Assessment of Negative Symptoms (SANS)*. University of Iowa.

Andreasen, N. C. (1984). *Scale for the Assessment of Positive Symptoms (SAPS)*. University of Iowa.

Bhojraj, T. S., Francis, A. N., Montrose, D. M., & Keshavan, M. S. (2011). Grey matter and cognitive deficits in young relatives of schizophrenia patients. *Neuroimage*, *54 Suppl 1*, S287-292. https://doi.org/10.1016/j.neuroimage.2010.03.069

Bhojraj, T. S., Francis, A. N., Rajarethinam, R., Eack, S., Kulkarni, S., Prasad, K. M., Montrose, D. M., Dworakowski, D., Diwadkar, V., & Keshavan, M. S. (2009). Verbal fluency deficits and altered lateralization of language brain areas in individuals genetically predisposed to schizophrenia. *Schizophr Res*, *115*(2-3), 202-208. https://doi.org/10.1016/j.schres.2009.09.033

Blokland, G. A. M., del Re, E. C., Mesholam-Gately, R. I., Jovicich, J., Trampush, J. W., Keshavan, M. S., DeLisi, L. E., Walters, J. T. R., Turner, J. A., Malhotra, A. K., Lencz, T., Shenton, M. E., Voineskos, A. N., Rujescu, D., Giegling, I., Kahn, R. S., Roffman, J. L., Holt, D. J., Ehrlich, S., . . . Petryshen, T. L. (2018). The Genetics of Endophenotypes of Neurofunction to Understand Schizophrenia (GENUS) consortium: A collaborative cognitive and neuroimaging genetics project. *Schizophr Res*, *195*, 306-317. https://doi.org/10.1016/j.schres.2017.09.024

Boos, H. B., Mandl, R. C., van Haren, N. E., Cahn, W., van Baal, G. C., Kahn, R. S., & Hulshoff Pol, H. E. (2013). Tract-based diffusion tensor imaging in patients with schizophrenia and their non-psychotic siblings. *Eur Neuropsychopharmacol*, *23*(4), 295-304. https://doi.org/10.1016/j.euroneuro.2012.05.015

Buka, S. L., Seidman, L. J., Tsuang, M. T., & Goldstein, J. M. (2013). The New England Family Study High-risk Project: neurological impairments among offspring of parents with schizophrenia and other psychoses. *Am J Med Genet B Neuropsychiatr Genet*, *162B*(7), 653-660. https://doi.org/10.1002/ajmg.b.32181

Clemm von Hohenberg, C., Pasternak, O., Kubicki, M., Ballinger, T., Vu, M. A., Swisher, T., Green, K., Giwerc, M., Dahlben, B., Goldstein, J. M., Woo, T. U., Petryshen, T. L., Mesholam-Gately, R. I., Woodberry, K. A., Thermenos, H. W., Mulert, C., McCarley, R. W., Seidman, L. J., & Shenton, M. E. (2014). White matter microstructure in individuals at clinical high risk of psychosis: A whole-brain diffusion tensor imaging study. *Schizophr Bull*, *40*(4), 895-903. https://doi.org/10.1093/schbul/sbt079

del Re, E. C., Bergen, S. E., Mesholam-Gately, R. I., Niznikiewicz, M. A., Goldstein, J. M., Woo, T. U., Shenton, M. E., Seidman, L. J., McCarley, R. W., & Petryshen, T. L. (2014). Analysis of schizophrenia-related genes and electrophysiological measures reveals ZNF804A association with amplitude of P300b elicited by novel sounds. *Transl Psychiatry*, *4*, e346. https://doi.org/10.1038/tp.2013.117

Donohoe, G., Walters, J., Morris, D. W., Quinn, E. M., Judge, R., Norton, N., Giegling, I., Hartmann, A. M., Moller, H. J., Muglia, P., Williams, H., Moskvina, V., Peel, R., O'Donoghue, T., Owen, M. J., O'Donovan, M. C., Gill, M., Rujescu, D., & Corvin, A. (2009). Influence of NOS1 on verbal intelligence and working memory in both patients with schizophrenia and healthy control subjects. *Arch Gen Psychiatry*, *66*(10), 1045-1054. https://doi.org/10.1001/archgenpsychiatry.2009.139

Francis, A. N., Seidman, L. J., Jabbar, G. A., Mesholam-Gately, R., Thermenos, H. W., Juelich, R., Proal, A. C., Shenton, M., Kubicki, M., Mathew, I., Keshavan, M., & Delisi, L. E. (2012). Alterations in brain structures underlying language function in young adults at high familial risk for schizophrenia. *Schizophr Res*, *141*(1), 65-71. https://doi.org/10.1016/j.schres.2012.07.015

Gardner, D. M., Murphy, A. L., O'Donnell, H., Centorrino, F., & Baldessarini, R. J. (2010). International consensus study of antipsychotic dosing. *Am J Psychiatry*, *167*(6), 686-693. https://doi.org/10.1176/appi.ajp.2009.09060802

Goldstein, J. M., Buka, S. L., Seidman, L. J., & Tsuang, M. T. (2010). Specificity of familial transmission of schizophrenia psychosis spectrum and affective psychoses in the New England Family Sudy's high-risk design. *Arch Gen Psychiatry*, *67*(5), 458-467. https://doi.org/10.1001/archgenpsychiatry.2010.38

Goldstein, J. M., Cherkerzian, S., Seidman, L. J., Donatelli, J. A., Remington, A. G., Tsuang, M. T., Hornig, M., & Buka, S. L. (2014). Prenatal maternal immune disruption and sex-dependent risk for psychoses. *Psychol Med*, *44*(15), 3249-3261. https://doi.org/10.1017/S0033291714000683

Gollub, R. L., Shoemaker, J. M., King, M. D., White, T., Ehrlich, S., Sponheim, S. R., Clark, V. P., Turner, J. A., Mueller, B. A., Magnotta, V., O'Leary, D., Ho, B. C., Brauns, S., Manoach, D. S., Seidman, L., Bustillo, J. R., Lauriello, J., Bockholt, J., Lim, K. O., . . . Andreasen, N. C. (2013). The MCIC collection: A shared repository of multi-modal, multi-site brain image data from a clinical investigation of schizophrenia. *Neuroinformatics*, *11*(3), 367-388. https://doi.org/10.1007/s12021-013-9184-3

Ho, N. F., Iglesias, J. E., Sum, M. Y., Kuswanto, C. N., Sitoh, Y. Y., De Souza, J., Hong, Z., Fischl, B., Roffman, J. L., Zhou, J., Sim, K., & Holt, D. J. (2016). Progression from selective to general involvement of hippocampal subfields in schizophrenia. *Mol Psychiatry*, *22*(1), 142-152. https://doi.org/10.1038/mp.2016.4

Holt, D. J., Coombs, G., Zeidan, M. A., Goff, D. C., & Milad, M. R. (2012). Failure of neural responses to safety cues in schizophrenia. *Arch Gen Psychiatry*, *69*(9), 893-903. https://doi.org/10.1001/archgenpsychiatry.2011.2310

Hulshoff Pol, H. E., Schnack, H. G., Mandl, R. C., van Haren, N. E., Koning, H., Collins, D. L., Evans, A. C., & Kahn, R. S. (2001). Focal gray matter density changes in schizophrenia. *Arch Gen Psychiatry*, *58*(12), 1118-1125. https://doi.org/10.1001/archpsyc.58.12.1118

Kay, S. R., Fiszbein, A., & Opler, L. A. (1987). The Positive And Negative Syndrome Scale (PANSS) for schizophrenia. *Schizophr Bull*, *13*(2), 261-276. https://doi.org/10.1093/schbul/13.2.261

Kikinis, Z., Fallon, J. H., Niznikiewicz, M., Nestor, P., Davidson, C., Bobrow, L., Pelavin, P. E., Fischl, B., Yendiki, A., McCarley, R. W., Kikinis, R., Kubicki, M., & Shenton, M. E. (2010). Gray matter volume reduction in rostral middle frontal gyrus in patients with chronic schizophrenia. *Schizophr Res*, *123*(2-3), 153-159. https://doi.org/10.1016/j.schres.2010.07.027

Kikinis, Z., Fitzsimmons, J., Dunn, C., Vu, M. A., Makris, N., Bouix, S., Goldstein, J. M., Mesholam-Gately, R. I., Petryshen, T., Del Re, E. C., Wojcik, J., Seidman, L. J., & Kubicki, M. (2015). Anterior commissural white matter fiber abnormalities in first-episode psychosis: A tractography study. *Schizophr Res*, *162*(1-3), 29-34. https://doi.org/10.1016/j.schres.2015.01.037

Korver, N., Quee, P. J., Boos, H. B., Simons, C. J., de Haan, L., & GROUP investigators. (2012). Genetic Risk and Outcome of Psychosis (GROUP), a multi-site longitudinal cohort study focused on gene-environment interaction: Objectives, sample characteristics, recruitment and assessment methods. *Int J Methods Psychiatr Res*, *21*(3), 205-221. https://doi.org/10.1002/mpr.1352

Kuswanto, C. N., Sum, M. Y., Qiu, A., Sitoh, Y. Y., Liu, J., & Sim, K. (2015). The impact of genome wide supported microRNA-137 (MIR137) risk variants on frontal and striatal white matter integrity, neurocognitive functioning, and negative symptoms in schizophrenia. *Am J Med Genet B Neuropsychiatr Genet*, *168B*(5), 317-326. https://doi.org/10.1002/ajmg.b.32314

Linnman, C., Coombs, G., 3rd, Goff, D. C., & Holt, D. J. (2013). Lack of insula reactivity to aversive stimuli in schizophrenia. *Schizophr Res*, *143*(1), 150-157. https://doi.org/10.1016/j.schres.2012.10.038

Nazeri, A., Chakravarty, M. M., Felsky, D., Lobaugh, N. J., Rajji, T. K., Mulsant, B. H., & Voineskos, A. N. (2013). Alterations of superficial white matter in schizophrenia and relationship to cognitive performance. *Neuropsychopharmacology*, *38*(10), 1954-1962. https://doi.org/10.1038/npp.2013.93

Nelson, H. E. (1982). *The National Adult Reading Test (NART): Test Manual*. NFER-Nelson.

O'Connor, J. A., Wiffen, B., Diforti, M., Ferraro, L., Joseph, C., Kolliakou, A., Bonaccorso, S., Murray, R. M., & David, A. S. (2013). Neuropsychological, clinical and cognitive insight predictors of outcome in a first episode psychosis study. *Schizophr Res*, *149*(1-3), 70-76. https://doi.org/10.1016/j.schres.2013.06.005

O'Donovan, M. C., Craddock, N., Norton, N., Williams, H., Peirce, T., Moskvina, V., Nikolov, I., Hamshere, M., Carroll, L., Georgieva, L., Dwyer, S., Holmans, P., Marchini, J. L., Spencer, C. C., Howie, B., Leung, H. T., Hartmann, A. M., Moller, H. J., Morris, D. W., . . . Molecular Genetics of Schizophrenia Collaboration. (2008). Identification of loci associated with schizophrenia by genome-wide association and follow-up. *Nat Genet*, *40*(9), 1053-1055. https://doi.org/10.1038/ng.201

Owens, S. F., Picchioni, M. M., Ettinger, U., McDonald, C., Walshe, M., Schmechtig, A., Murray, R. M., Rijsdijk, F., & Toulopoulou, T. (2012). Prefrontal deviations in function but not volume are putative endophenotypes for schizophrenia. *Brain*, *135*(Pt 7), 2231-2244. https://doi.org/10.1093/brain/aws138

Owens, S. F., Picchioni, M. M., Rijsdijk, F. V., Stahl, D., Vassos, E., Rodger, A. K., Collier, D. A., Murray, R. M., & Toulopoulou, T. (2011). Genetic overlap between episodic memory deficits and schizophrenia: Results from the Maudsley Twin Study. *Psychol Med*, *41*(3), 521-532. https://doi.org/10.1017/S0033291710000942

Owens, S. F., Rijsdijk, F., Picchioni, M. M., Stahl, D., Nenadic, I., Murray, R. M., & Toulopoulou, T. (2011). Genetic overlap between schizophrenia and selective components of executive function. *Schizophr Res*, *127*(1-3), 181-187. https://doi.org/10.1016/j.schres.2010.10.010

Pasternak, O., Westin, C. F., Bouix, S., Seidman, L. J., Goldstein, J. M., Woo, T. U., Petryshen, T. L., Mesholam-Gately, R. I., McCarley, R. W., Kikinis, R., Shenton, M. E., & Kubicki, M. (2012). Excessive extracellular volume reveals a neurodegenerative pattern in schizophrenia onset. *J Neurosci*, *32*(48), 17365-17372. https://doi.org/10.1523/JNEUROSCI.2904-12.2012

Psychosis Endophenotypes International Consortium, Wellcome Trust Case-Control Consortium, Bramon, E., Pirinen, M., Strange, A., Lin, K., Freeman, C., Bellenguez, C., Su, Z., Band, G., Pearson, R., Vukcevic, D., Langford, C., Deloukas, P., Hunt, S., Gray, E., Dronov, S., Potter, S. C., Tashakkori-Ghanbaria, A., . . . Spencer, C. C. (2014). A genome-wide association analysis of a broad psychosis phenotype identifies three loci for further investigation. *Biol Psychiatry*, *75*(5), 386-397. https://doi.org/10.1016/j.biopsych.2013.03.033

Quan, M., Lee, S. H., Kubicki, M., Kikinis, Z., Rathi, Y., Seidman, L. J., Mesholam-Gately, R. I., Goldstein, J. M., McCarley, R. W., Shenton, M. E., & Levitt, J. J. (2013). White matter tract abnormalities between rostral middle frontal gyrus, inferior frontal gyrus and striatum in first-episode schizophrenia. *Schizophr Res*, *145*(1-3), 1-10. https://doi.org/10.1016/j.schres.2012.11.028

Rais, M., Cahn, W., Schnack, H. G., Hulshoff Pol, H. E., Kahn, R. S., & van Haren, N. E. (2012). Brain volume reductions in medication-naive patients with schizophrenia in relation to intelligence quotient. *Psychol Med*, *42*(9), 1847-1856. https://doi.org/10.1017/S0033291712000098

Reis Marques, T., Taylor, H., Chaddock, C., Dell'acqua, F., Handley, R., Reinders, A. A., Mondelli, V., Bonaccorso, S., Diforti, M., Simmons, A., David, A. S., Murray, R. M., Pariante, C. M., Kapur, S., & Dazzan, P. (2014). White matter integrity as a predictor of response to treatment in first episode psychosis. *Brain*, *137*(Pt 1), 172-182. https://doi.org/10.1093/brain/awt310

Roffman, J. L., Lamberti, J. S., Achtyes, E., Macklin, E. A., Galendez, G. C., Raeke, L. H., Silverstein, N. J., Smoller, J. W., Hill, M., & Goff, D. C. (2013). Randomized multicenter investigation of folate plus vitamin B12 supplementation in schizophrenia. *JAMA Psychiatry*, *70*(5), 481-489. https://doi.org/10.1001/jamapsychiatry.2013.900

Roffman, J. L., Nitenson, A. Z., Agam, Y., Isom, M., Friedman, J. S., Dyckman, K. A., Brohawn, D. G., Smoller, J. W., Goff, D. C., & Manoach, D. S. (2011). A hypomethylating variant of MTHFR, 677C>T, blunts the neural response to errors in patients with schizophrenia and healthy individuals. *PLoS One*, *6*(9), e25253. https://doi.org/10.1371/journal.pone.0025253

Roffman, J. L., Weiss, A. P., Deckersbach, T., Freudenreich, O., Henderson, D. C., Purcell, S., Wong, D. H., Halsted, C. H., & Goff, D. C. (2007). Effects of the methylenetetrahydrofolate reductase (MTHFR) C677T polymorphism on executive function in schizophrenia. *Schizophr Res*, *92*(1-3), 181-188. https://doi.org/10.1016/j.schres.2007.01.003

Roffman, J. L., Weiss, A. P., Deckersbach, T., Freudenreich, O., Henderson, D. C., Wong, D. H., Halsted, C. H., & Goff, D. C. (2008). Interactive effects of COMT Val108/158Met and MTHFR C677T on executive function in schizophrenia. *Am J Med Genet B Neuropsychiatr Genet*, *147B*(6), 990-995. https://doi.org/10.1002/ajmg.b.30684

Roffman, J. L., Weiss, A. P., Purcell, S., Caffalette, C. A., Freudenreich, O., Henderson, D. C., Bottiglieri, T., Wong, D. H., Halsted, C. H., & Goff, D. C. (2008). Contribution of methylenetetrahydrofolate reductase (MTHFR) polymorphisms to negative symptoms in schizophrenia. *Biol Psychiatry*, *63*(1), 42-48. https://doi.org/10.1016/j.biopsych.2006.12.017

Seidman, L. J., Cherkerzian, S., Goldstein, J. M., Agnew-Blais, J., Tsuang, M. T., & Buka, S. L. (2013). Neuropsychological performance and family history in children at age 7 who develop adult schizophrenia or bipolar psychosis in the New England Family Studies. *Psychol Med*, *43*(1), 119-131. https://doi.org/10.1017/S0033291712000773

Seidman, L. J., Kremen, W. S., Koren, D., Faraone, S. V., Goldstein, J. M., & Tsuang, M. T. (2002). A comparative profile analysis of neuropsychological functioning in patients with schizophrenia and bipolar psychoses. *Schizophr Res*, *53*(1-2), 31-44. https://doi.org/10.1016/s0920-9964(01)00162-1

Seitz, J., Zuo, J. X., Lyall, A. E., Makris, N., Kikinis, Z., Bouix, S., Pasternak, O., Fredman, E., Duskin, J., Goldstein, J. M., Petryshen, T. L., Mesholam-Gately, R. I., Wojcik, J., McCarley, R. W., Seidman, L. J., Shenton, M. E., Koerte, I. K., & Kubicki, M. (2016). Tractography analysis of 5 white matter bundles and their clinical and cognitive correlates in early-course schizophrenia. *Schizophr Bull*, *42*(3), 762-771. https://doi.org/10.1093/schbul/sbv171

Terwisscha van Scheltinga, A. F., Bakker, S. C., van Haren, N. E., Buizer-Voskamp, J. E., Boos, H. B., Vorstman, J., Cahn, W., Hulshoff Pol, H., Ophoff, R. A., & Kahn, R. S. (2012). Association study of copy number variants with brain volume in schizophrenia patients and healthy controls. *Psychiatry Res*, *200*(2-3), 1011-1013. https://doi.org/10.1016/j.psychres.2012.04.007

Terwisscha van Scheltinga, A. F., Bakker, S. C., van Haren, N. E., Derks, E. M., Buizer-Voskamp, J. E., Boos, H. B., Cahn, W., Hulshoff Pol, H. E., Ripke, S., Ophoff, R. A., Kahn, R. S., & Psychiatric Genome-wide Association Study Consortium. (2013). Genetic schizophrenia risk variants jointly modulate total brain and white matter volume. *Biol Psychiatry*, *73*(6), 525-531. https://doi.org/10.1016/j.biopsych.2012.08.017

Terwisscha van Scheltinga, A. F., Bakker, S. C., van Haren, N. E., Derks, E. M., Buizer-Voskamp, J. E., Cahn, W., Ripke, S., Psychiatric Genome-Wide Association Study Consortium, Ophoff, R. A., & Kahn, R. S. (2013). Schizophrenia genetic variants are not associated with intelligence. *Psychol Med*, *43*(12), 2563-2570. https://doi.org/10.1017/S0033291713000196

Theleritis, C., Fisher, H. L., Shafer, I., Winters, L., Stahl, D., Morgan, C., Dazzan, P., Breedvelt, J., Sambath, I., Vitoratou, S., Russo, M., Reichenberg, A., Falcone, M. A., Mondelli, V., O'Connor, J., David, A., McGuire, P., Pariante, C., Di Forti, M., . . . Bonaccorso, S. (2014). Brain derived Neurotropic Factor (BDNF) is associated with childhood abuse but not cognitive domains in first episode psychosis. *Schizophr Res*, *159*(1), 56-61. https://doi.org/10.1016/j.schres.2014.07.013

Toulopoulou, T., Picchioni, M., Rijsdijk, F., Hua-Hall, M., Ettinger, U., Sham, P., & Murray, R. (2007). Substantial genetic overlap between neurocognition and schizophrenia: Genetic modeling in twin samples. *Arch Gen Psychiatry*, *64*(12), 1348-1355. https://doi.org/10.1001/archpsyc.64.12.1348

van Erp, T. G., Preda, A., Nguyen, D., Faziola, L., Turner, J., Bustillo, J., Belger, A., Lim, K. O., McEwen, S., Voyvodic, J., Mathalon, D. H., Ford, J., Potkin, S. G., & Fbirn. (2014). Converting positive and negative symptom scores between PANSS and SAPS/SANS. *Schizophr Res*, *152*(1), 289-294. https://doi.org/10.1016/j.schres.2013.11.013

Vassos, E., Di Forti, M., Coleman, J., Iyegbe, C., Prata, D., Euesden, J., O'Reilly, P., Curtis, C., Kolliakou, A., Patel, H., Newhouse, S., Traylor, M., Ajnakina, O., Mondelli, V., Marques, T. R., Gardner-Sood, P., Aitchison, K. J., Powell, J., Atakan, Z., . . . Breen, G. (2017). An examination of polygenic score risk prediction in individuals with first-episode psychosis. *Biol Psychiatry*, *81*(6), 470-477. https://doi.org/10.1016/j.biopsych.2016.06.028

Walters, J. T., Corvin, A., Owen, M. J., Williams, H., Dragovic, M., Quinn, E. M., Judge, R., Smith, D. J., Norton, N., Giegling, I., Hartmann, A. M., Moller, H. J., Muglia, P., Moskvina, V., Dwyer, S., O'Donoghue, T., Morar, B., Cooper, M., Chandler, D., . . . Donohoe, G. (2010). Psychosis susceptibility gene ZNF804A and cognitive performance in schizophrenia. *Arch Gen Psychiatry*, *67*(7), 692-700. https://doi.org/10.1001/archgenpsychiatry.2010.81

Walters, J. T., Rujescu, D., Franke, B., Giegling, I., Vasquez, A. A., Hargreaves, A., Russo, G., Morris, D. W., Hoogman, M., Da Costa, A., Moskvina, V., Fernandez, G., Gill, M., Corvin, A., O'Donovan, M. C., Donohoe, G., & Owen, M. J. (2013). The role of the major histocompatibility complex region in cognition and brain structure: A schizophrenia GWAS follow-up. *Am J Psychiatry*, *170*(8), 877-885. https://doi.org/10.1176/appi.ajp.2013.12020226

Wechsler, D. (1974). *Wechsler Adult Intelligence Scale–Revised (WAIS-R)*. The Psychological Corporation.

Wechsler, D. (1981). *Examiner's Manual: Wechsler Adult Intelligence Scale–Revised (WAIS-R)*. The Psychological Corporation.

Wechsler, D. (1997). *Wechsler Adult Intelligence Scale–Third Edition (WAIS-III)*. The Psychological Corporation.

Wechsler, D. (1999). *Wechsler Abbreviated Scale of Intelligence (WASI)*. The Psychological Corporation.

Wechsler, D. (2001). *Wechsler Test of Adult Reading*. Harcourt Assessment.

Wechsler, D. (2005). *Wechsler Adult Intelligence Scale-Third Edition, UK Edition (WAIS-III-UK)*. Psychological Corporation Ltd.

Wheeler, A. L., Chakravarty, M. M., Lerch, J. P., Pipitone, J., Daskalakis, Z. J., Rajji, T. K., Mulsant, B. H., & Voineskos, A. N. (2014). Disrupted prefrontal interhemispheric structural coupling in schizophrenia related to working memory performance. *Schizophr Bull*, *40*(4), 914-924. https://doi.org/10.1093/schbul/sbt100

Wilkinson, G. S. (1993). *Wide Range Achievement Test–Third Edition (WRAT-3)*. Jastak Association.

Wilkinson, G. S., & Robertson, G. R. (2006). *Wide Range Achievement Test 4 (WRAT-4) Professional Manual*. Psychological Assessment Resources.

Woods, S. W. (2003). Chlorpromazine equivalent doses for the newer atypical antipsychotics. *J Clin Psychiatry*, *64*(6), 663-667. https://doi.org/10.4088/jcp.v64n0607
